# Selective modulation of the glucocorticoid receptor can distinguish between transrepression of NF-κB and AP-1

**DOI:** 10.1007/s00018-013-1367-4

**Published:** 2013-06-20

**Authors:** Karolien De Bosscher, Ilse M. Beck, Lien Dejager, Nadia Bougarne, Anthoula Gaigneaux, Sébastien Chateauvieux, Dariusz Ratman, Marc Bracke, Jan Tavernier, Wim Vanden Berghe, Claude Libert, Marc Diederich, Guy Haegeman

**Affiliations:** 1 Laboratory of Eukaryotic Gene Expression & Signal Transduction (LEGEST), Department of Physiology, Ghent University, Ghent, Belgium; 2 Laboratory of Experimental Cancer Research, Department Radiation Therapy and Experimental Cancer Research, Ghent University Hospital, De Pintelaan 185, 1P7, 9000, Ghent, Belgium; 3 Cytokine Receptor Lab, VIB Department of Medical Protein Research, Ghent University, Albert Baertsoenkaai 3, 9000 Ghent, Belgium; 4 Department for Molecular Biomedical Research, VIB, Ghent, Belgium; 5 Department of Biomedical Molecular Biology, Ghent University, Ghent, Belgium; 6 Laboratoire de Biologie Moléculaire et Cellulaire du Cancer (LBMCC), Hôpital Kirchberg, Luxemburg, Luxemburg; 7 Lab Protein Chemistry, Proteomics and Epigenetic Signaling (PPES), Department Biomedical Sciences, University of Antwerp, Antwerpen, Belgium; 8 Department of Pharmacy, College of Pharmacy, Seoul National University, Seoul, 151-742 South Korea; 9 Present Address: Division of Molecular Medicine, Department of Research and Development, Faculty of Medicine, Siriraj Hospital, Mahidol University, Bangkok, 10700 Thailand

**Keywords:** Glucocorticoids, Inflammation, Mitogen-activated protein kinase (MAPK), Selective GR modulator, c-Jun, Jun N-terminal kinase (JNK)

## Abstract

Glucocorticoids (GCs) block inflammation via interference of the liganded glucocorticoid receptor (GR) with the activity of pro-inflammatory transcription factors NF-κB and AP-1, a mechanism known as transrepression. This mechanism is believed to involve the activity of GR monomers. Here, we explored how the GR monomer-favoring Compound A (CpdA) affects AP-1 activation and activity. Our results demonstrate that non-steroidal CpdA, unlike classic steroidal GCs, blocks NF-κB- but not AP-1-driven gene expression. CpdA rather sustains AP-1-driven gene expression, a result which could mechanistically be explained by the failure of CpdA to block upstream JNK kinase activation and concomitantly also phosphorylation of c-Jun. In concordance and in contrast to DEX, CpdA maintained the expression of the activated AP-1 target gene *c*-*jun*, as well as the production of the c-Jun protein. As for the underlying mechanism, GR is a necessary intermediate in the CpdA-mediated gene expression of AP-1-regulated genes, but seems to be superfluous to CpdA-mediated JNK phosphorylation prolongation. The latter phenomenon concurs with the inability of CpdA to stimulate DUSP1 gene expression. ChIP analysis demonstrates that DEX-activated GR, but not CpdA-activated GR, is recruited to AP-1-driven promoters. Furthermore, in mice we observed that CpdA instigates a strong enhancement of TNF-induced AP-1-driven gene expression. Finally, we demonstrate that this phenomenon coincides with an increased sensitivity towards TNF lethality, and implicate again a role for JNK2. In conclusion, our data support the hypothesis that a ligand-induced differential conformation of GR yields a different transcription factor cross-talk profile.

**Electronic supplementary material:**

The online version of this article (doi:10.1007/s00018-013-1367-4) contains supplementary material, which is available to authorized users.

## Introduction

The glucocorticoid receptor (GR, NR3C1) is a ligand-dependent transcription factor belonging to the subfamily 3C of the nuclear receptor superfamily [1]. GR comprises a N-terminal transactivation domain, a DNA-binding domain (DBD) and a C-terminal ligand-binding domain (LBD) [2, 3]. Classic GR target gene promoter activation occurs via interaction of homodimeric GR with specific GR-binding sequences (GBS) at glucocorticoid response elements (GRE) [4–6]. GR can also orchestrate transcriptional networks via protein–protein interactions, resulting in a positive or negative transcriptional outcome, depending on the target gene and the cellular context [7]. The complex mechanisms by which glucocorticoids (GCs) inhibit gene expression, of which transrepression has been best described, have always received the most attention, because they allow to explain a large part of the immunosuppressive action of GCs [6, 8].

The promoters of various genes coding for proteins involved in inflammatory processes, including cytokines, chemokines, and adhesion molecules harbor specific DNA sequences onto which the pro-inflammatory transcription factors NF-κB and/or AP-1 can bind. NF-κB, typically a heterodimer of p65 (RelA) and p50 (NF-κB1) Rel family members, predominantly resides in the cytoplasm in complex with IκB inhibitory proteins. Activation of the IKK complex leads it to phosphorylate the IκB inhibitory protein, marking it for subsequent degradation. Hence, NF-κB is released from its inhibitor, allowing its migration to the nucleus. Mitogen-activated protein kinases (MAPKs), ERK and p38, and downstream MAPK-activated proteins, including MSK1 additionally fine-tune its activity [9]. Various stimuli, including cytokines (such as TNF-α) [10] and the microbial alkaloid staurosporine (STS) [11] result in the activation of nuclear NF-κB and/or AP-1, which contact their regulatory DNA sequences and, as such, drive gene transcription of, e.g., the cytokine Interleukin-6 (IL-6) [12]. This cytokine has not only been implicated in immune regulation but also in endocrine and metabolic actions and aging. Understanding the regulation of this gene could contribute to a controlled and tissue-restricted modulation of its pleiotropic action profile.

The *c*-*jun* proto-oncogene codes for c-Jun, which constitutes together with c-Fos the prototypical dimeric AP-1 transcription factor [13, 14]. Many pro-inflammatory genes are co-regulated by NF-κB and AP-1. However, an immediate early gene such as *c*-*jun*, is regulated by AP-1 alone, and not by NF-κB. The activation of the c-Jun protein is rapidly regulated in response to a wide variety of external stimuli, including cytokines, tumor-promoting agents, UV irradiation, growth factors and hormones, and it does not require de novo protein synthesis [13, 15, 16]. Notably, AP-1 can bind to the *c*-*jun* gene promoter itself, thereby stimulating gene transcription via a feed-forward mechanism [17]. Two regulatory AP-1 binding elements have been described in the *c*-*jun* gene promoter, a proximal one and a distal one [17, 18]. Both AP-1 sites have been found to be susceptible to GR-mediated transrepression [15]. The Jun N-terminal kinase JNK is the most prominent MAPK involved in the regulation of AP-1 [19]. Phosphorylation by JNK rapidly potentiates the transcriptional capacity of c-Jun, enhancing its ability to accommodate gene transcription, including its own [19]. In that respect, interactions between AP-1 and GC signaling pathways are not restricted to direct transcriptional interferences between GR and AP-1 [20]; GCs can also target the activity of JNK, which can be stimulated by pro-inflammatory cytokines, including TNF-α [21, 22].

Glucocorticoids (GCs) remain the gold standard in the treatment of chronic inflammatory diseases not only because they can efficiently relieve the inflammation-associated symptoms, but also because they act as disease-modifiers [23]. Mechanistically, many of the anti-inflammatory effects of GCs can be traced back to their gene-repressive effect, targeting GR to key transcription factors which otherwise drive various inflammatory factors. However, upon chronic exogenous GC treatment, the associated side effects, such as diabetes, osteoporosis, and skin bruising and thinning, remain cumbersome [24]. In that respect, insulin resistance, and diabetes in particular, and also other side effects, are considered to arise mainly from the transactivation function of GR. Consequently, the impetus to develop novel selective GR modulators (SGRM) has never been stronger [25, 26]. Dissociating GR functionalities to improve therapeutic benefit is a concept that has furthermore been supported by gene-targeting experiments: transgenic mice with a dimerization-defective GR deficient in DNA binding still demonstrate functional transrepression and a GC-mediated anti-inflammatory response [27, 28]. Synthetic steroidal ligands for GR allowing a separation of GR-dependent transactivation and transrepression capacities in vitro, have not always maintained this characteristic in vivo [29]. In contrast, non-steroidal GR ligands, including AL-438, ZK216348, ZK245186, LGD5552, and Compound A (CpdA), have met these requirements with greater success in inflammatory animal model studies, although only a few of those have passed the pre-clinical stage (reviewed in [25, 26]).

Using genetic mouse models, a role for JNK2 activity, as controlled via a GR dimerization-dependent mechanism, has recently been implicated in the protection against systemic TNF-induced lethal inflammation [30]. This finding indicates that a selection towards GR-mediated monomerization might not always be beneficial, and supports a contributory role for GC-induced anti-inflammatory proteins, including MAPK phosphatase MKP-1 (encoded by the *Dusp1* gene) in resolving inflammation in vivo [30]. On the other hand, the recent finding that dimerization-defective GR mutants could still retain dimerization capacities in vitro questions the extent of the receptor’s dissociative properties and hence challenges the transactivation versus transrepression model [31, 32]. However, it is as yet unclear to what extent and onto which specific promoters a dimerization may still proceed in vivo. Nonetheless, an attempt to favor immuno-modulatory effects over the potential scala of side effects, the restriction of GR signaling to well-defined pathways remains a valid strategy. As such, the exploration of differences and parallels between the GR-mediated transrepression of key inflammatory transcription factors, such as NF-κB and AP-1, is an important research area.

## Materials and methods

### Cell culture

Murine L929sA fibrosarcoma cells were maintained in DMEM (Gibco-Invitrogen, Merelbeke, Belgium) supplemented with 5 % fetal and 5 % newborn calf serum (International Medical Products, Brussels, Belgium), while human A549 lung epithelial cells were maintained in DMEM supplemented with 10 % fetal calf serum. To both culture media, 100 U/ml penicillin and 0.1 mg/ml streptomycin (Sigma-Aldrich, St. Louis, MO, USA) was added.

### Mice

C57BL6/J mice were purchased from Janvier (Le Genest-St Isle, France). JNK-2^−/−^ mice had a C57BL6/J background and were purchased from the Jackson Laboratory (Bar Harbor, MA, USA). Mice were kept in individually ventilated cages under a dark-light cycle of 12 h each in a conventional animal house and received food and water ad libitum. All mice were used at the age of 8–12 weeks.

### Plasmids

The full-size IL-6 promoter reporter gene construct p1168hu.IL6P-luc and the point-mutated variant p1168(AP-1 mut).IL6P-luc were previously described [33]. The reporter gene plasmid pAP1-luc was purchased from Stratagene Cloning Systems (La Jolla, CA, USA). The reporter gene plasmid p(IL6-κB)_3_-50hu.IL6P-luc has been described before [34] and the β-Gal-expressing plasmid to control for transfection efficiencies in transient transfection assays and/or cellular viability upon inductions was a kind gift from Dr. A. Liberman (University of Buenos Aires, Buenos Aires, Argentina). The pCollagenase-3-luc reporter gene plasmid was a kind gift from Dr. E. Canalis (Saint Francis Hospital and Medical Center, Hartford, Connecticut, USA).

### Cytokines, reagents, and antibodies

Dexamethasone (DEX) and 12-*O*-tetradecanoylphorbol-13-acetate (TPA) were purchased from Sigma-Aldrich. Recombinant mouse TNF was produced in *E. coli* and purified to homogeneity in our laboratories. TNF had a specific activity of 1.2 × 10^8^ IU/mg and had no detectable endotoxin contamination. The preparation of luciferase (luc) reagent was described previously [33]. The origin, handling and storage of CpdA was previously described [35]. Staurosporine (STS) was purchased from Calbiochem–Novabiochem International (San Diego, CA, USA). Luciferase (luc) assays were carried out according to the protocol of Promega Corp. (Madison, WI, USA). Control experiments showed that the final quantities of organic solvent used did not interfere with any of the assays. Normalization of luc activity, expressed as arbitrary light units, was performed by measurement of β-galactosidase (β-gal) levels in a chemi-luminescent reporter assay Galacto-Light kit (Tropix, Inc., Bedford, MA, USA). Light emission was measured in a luminescence microplate counter Victor Wallac (Perkin-Elmer, Cambridge, UK).

The phospho-specific p38 (Thr-180/Tyr-182), p42/p44 (Thr-202/Tyr-204), and SAPK/JNK (Thr-183/Tyr-185) MAPK polyclonal rabbit antibodies detecting only the dual phosphorylated form of MAPK, and their non-phospho-counterparts were purchased from Cell Signaling Technology (Beverly, MA, USA). The same company also supplied the phospho-c-Jun (Ser-73) antibody and the anti-rabbit and anti-mouse IgG coupled to horseradish peroxidase, the latter of which was used as a second antibody for Western blotting. Additional secondary antibodies, Goat anti-rabbit IgG (H + L) Dylight 800 conjugated (#35571) and Goat anti-mouse IgG (H + L) Dylight 680 conjugated (#35518) to use for development with the Odyssey (LI-COR, Lincoln, NE, USA) were obtained from Thermo Scientific. The MAPK analyzes itself were performed as described before [36]. The NF-κB p65, IκBα, and c-Jun antibodies were purchased from Santa Cruz (Santa Cruz Biotechnology, Santa Cruz, CA, USA), the actin antibody was acquired via Sigma (Irvine, UK).

### Transfections

Stable transfections of L929sA cells were described previously [33]. L929sA cells were transiently transfected by a standard calcium phosphate coprecipitation protocol. Briefly, 10^5^ actively growing cells were seeded in a 24-well plate 24 h before transfection. At day 0, 400 ng of total DNA was transfected. Sixteen hours post-transfection the medium was replaced with fresh medium and cells were left to rest for another 24 h, after which inductions were performed as indicated in the figure legends. Cells were lysed with lysis buffer (Tropix, Inc., Bedford, MA, USA), and samples were assayed for their β-gal content and luciferase activity.

### Injections and sampling

For the mice experiments, TNF was diluted in pyrogen-free phosphate-buffered saline (PBS) and all injections were given intraperitoneally (i.p.). Blood was withdrawn with a glass capillary from the retro-orbital plexus and after clotting (overnight at 4 °C) serum was collected upon centrifugation. To sample liver tissue, mice were killed by cervical dislocation, liver was isolated and stored in RNA later (Qiagen Benelux bv., Venlo, The Netherlands) before RNA preparation using an RNeasy Mini kit (Qiagen Benelux bv., Venlo, The Netherlands) according to the manufacturer’s instructions. All animal experiments were approved by the institutional ethics committee for animal welfare at the Faculty of Sciences, Ghent University.

### GR knockdown

The targeting siRNA for GR knockdown species-specific siGR (siGENOMESMARTpool NR3C1) and non-targeting control (siControl) were purchased via Dharmacon (Thermo Fisher Scientific, Lafayette, CO, USA). The siRNA was transfected into L929sA cells and A549 cells as described previously [37, 38]. Subsequent to the indicated inductions, total RNA was isolated using TRIzol Reagent (Invitrogen, Carlsbad, CA,USA). RNA samples were analyzed via RT-qPCR as described below. Control protein samples of L929sA cells were analyzed via Western blot analysis, as described [37].

### qPCR analysis

Following the treatment, as described in the figure legend, total RNA was isolated using TRIzol reagent (Invitrogen, Carlsbad, CA, USA) following the manufacturer’s instructions. RNA concentrations of samples were determined and 500 ng RNA was used in a RT-step with MMLV reverse transcriptase (Promega, Madison, WI, USA) to produce the respective cDNA. Subsequently, the obtained cDNA was assayed for the gene expression levels of human GR, IL-6, IL-8, or *c*-*jun* (for A549 cells) or murine GR, IL-6, *c*-*jun,* TNF, DUSP1, MMP13, TIMP1, MCP1, IκBα or MCP-1 for L929sA cells and/or murine samples) and at least two household gene levels as determined via Genorm [39] via qPCR in an ICycler (Bio-Rad, Hercules, CA, USA) using Sopachem reagents (Sopachem, Eke, Belgium) or a Lightcycler 480 System using Lightcycler 480 SYBRGreen I Master reagents (Roche Diagnostics, Vilvoorde, Belgium). Primer sequences used are readily available upon request.

### Agilent array data analysis

After a starvation period of 24 h in DMEM devoid of serum, A549 cells were pretreated for 1 h with solvent, DEX (1 μM) or CpdA (10 μM), either or not followed by 3-h treatment with TNF (2,000 IU/ml). Total mRNA was isolated with TRIZOL (Invitrogen, Carlsbad, CA, USA) and purified with a RNeasy kit (Qiagen Benelux bv., Venlo, The Netherlands), according to the manufacturers’ instructions. cDNA was labeled and amplified to cRNA for hybridization with the LowInput QuickAmp Labeling Kit Two-Color from Agilent Technologies (Diegem, Belgium). The hybridized and washed probes on each glass slide were scanned by an Agilent DNA microarray scanner with Surescan High-Resolution Technology and digital data extracted by Agilent’s Feature Extraction software 10.7.1.1 (Agilent). All treatments were performed in triplicate. The control condition was considered as a common reference.

Gene expression data were analyzed using the BioConductor [40] package “LIMMA” (ver. 3.6.1) [41] in the R statistical programming environment (ver. 2.12.0) [42]. A quality-control step was performed to increase the power of differential expression analysis by identifying measures from lower reliability spots. Two measures were used as lower quality markers (i.e., “gIsPosAndSignif” column provided by Agilent scan software and the signal/noise ratio). Spots flagged as bad in every array were removed from the analysis.

Preprocessing was performed without background subtraction, as it was found to best keep correlation between replicates. Within-array normalization was performed using “loess” algorithm and between array normalization was performed using “Aquantile”. Entrez Gene IDs were assigned to the corresponding Agilent probe ID, using the Bioconductor annotation package “hgug4112a.db” ver. 2.4.5 [43]. Non-specific filtering was obtained by removing spots without an Entrez Gene ID annotation. In addition, spots that didn’t reach at least 3 times (i.e. one condition) an expression level of 150 were removed. This left 22446 probes for analysis.

A linear model was built for every condition and included a dye effect to account for any gene-wise dye bias. Contrasts were used to extract differently expressed genes between the following conditions: “A vs. Ctrl”, “TNF vs. Ctrl”, “A+TNF vs. Ctrl”, “A+TNF vs. TNF”, “Dex vs. Ctrl”, “Dex+TNF vs. Ctrl”, “Dex+TNF vs. TNF”, “A+TNF vs. Dex+TNF”. A correction for multiple hypothesis testing was performed on *p* values prior to gene selection using Benjamini and Hochberg’s algorithm. Significant spots were selected on the basis of a false discovery rate adjusted *p* value cut-off of 0.05. In case multiple spots on the microarray were related to the same gene, only the most significant spot, corresponding to the highest *F* statistic, was selected for further analysis. This resulted in 13,944 genes analyzed. The gene list for comparative analysis was built by selecting genes significant (*p* < 0.05) in at least one contrast of interest (including “TNF vs. Ctrl”, “DEX/TNF vs. TNF”, “CpdA/TNF vs. TNF” contrasts) and restricted to genes presenting a fold change of 1.3 in at least one contrast of interest.

For the generated gene lists, the promoter sequences (−450 bp to +50 bp) were analyzed for statistically significant (*p* < 0.05) overrepresented transcription factor binding motifs of the Jaspar database by Pscan [44–46] and displayed NF-κB and AP-1 family members. Additionally, the generated gene lists were also analyzed via ingenuity pathway analysis (IPA). Each group was used as an input set for the “Core Analysis” with default settings, except: Reference set = Genes only; Relationship = Direct; Confidence = Experimentally observed.

### Chromatin immunoprecipitation (ChIP)

L929sA cells were starved in 0 % DMEM for 48 h. After the appropriate inductions, cells were subjected to a ChIP assay using an antibody against the GR (H-300, Santa Cruz). The ChIP analysis itself was performed as previously described [47]. DNA was purified using a QiaQuick purification kit (Qiagen Benelux bv., Venlo, The Netherlands). The amount of sonicated protein–DNA complexes, present before immunoprecipitation (IP), is measured in the input controls. Purified DNA samples, enriched with the immunoprecipitated protein and input controls, were subjected to qPCR in triplicate. Subsequently, the data obtained for immunoprecipitated samples were corrected for the respective signal from input control. To allow ratio comparisons, relative recruitment (Bound/Input) of the Solvent condition was set at 1 and all other conditions were recalculated accordingly.

### ELISA

Murine IL-6 ELISA was performed using a kit from Biosource (Invitrogen, Merelbeke, Belgium) for cellular media. IL-6 protein levels in in vivo samples were assayed using a 7TD1 bioassay [48].

### Statistics

Statistical significance on averaged results of minimally two independent experiments was determined using one-way ANOVA tests followed by a Tukey multiple comparison post test or, for the in vivo analyses, via an unpaired* t* test. Survival curves (Kaplan–Meier plots) were compared by logrank test. Values as of *p* < 0.05 were considered significant.

## Results

### CpdA favors selective GR transrepression of NF-κB in the human IL-6 promoter

**Fig. 1:**
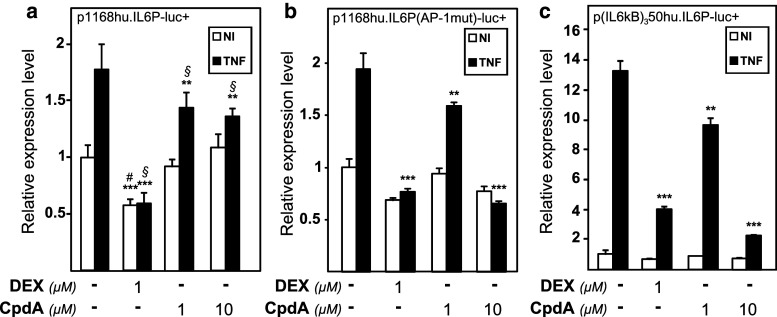
CpdA-mediated transrepression of the IL-6 gene promoter in fibroblast cells is only efficient in absence of a functional AP-1-response element. **a**, **b** Subconfluent L929sA cell monolayers stably transfected with the indicated promoter reporter gene constructs were grown in 24-well plates. The point-mutated variant is indicated by its mutated transcription factor-binding site, i.e., AP-1, between brackets. **c** Similar to panels (**a**) and (**b**), but with inductions performed on L929sA cells with a stably integrated recombinant (IL6κB)_3_50hu.IL6P-luc+ reporter construct. Cells were left untreated or treated with 2,000 IU/ml TNF, for 5 h, preceded by a 1 h treatment with solvent (as indicated by the* minus sign*), DEX (1 μM) or CpdA (1 or 10 μM). At the end of the induction, cell lysates were assayed for reporter gene activities. Total solvent concentration was kept similar in all conditions. The experiments are carried out in triplicate or quadruplicate. Results are shown ± SD and are representative of two to four independent experiments. **p < 0.01, ***p < 0.001. For (**a**) comparisons versus the control lane are depicted by ‘#’ and comparisons versus the respective pro-inflammatory stimulus TNF are depicted by ‘§’. For **b** and **c**, comparisons were made vs. TNF

**Fig. 2:**
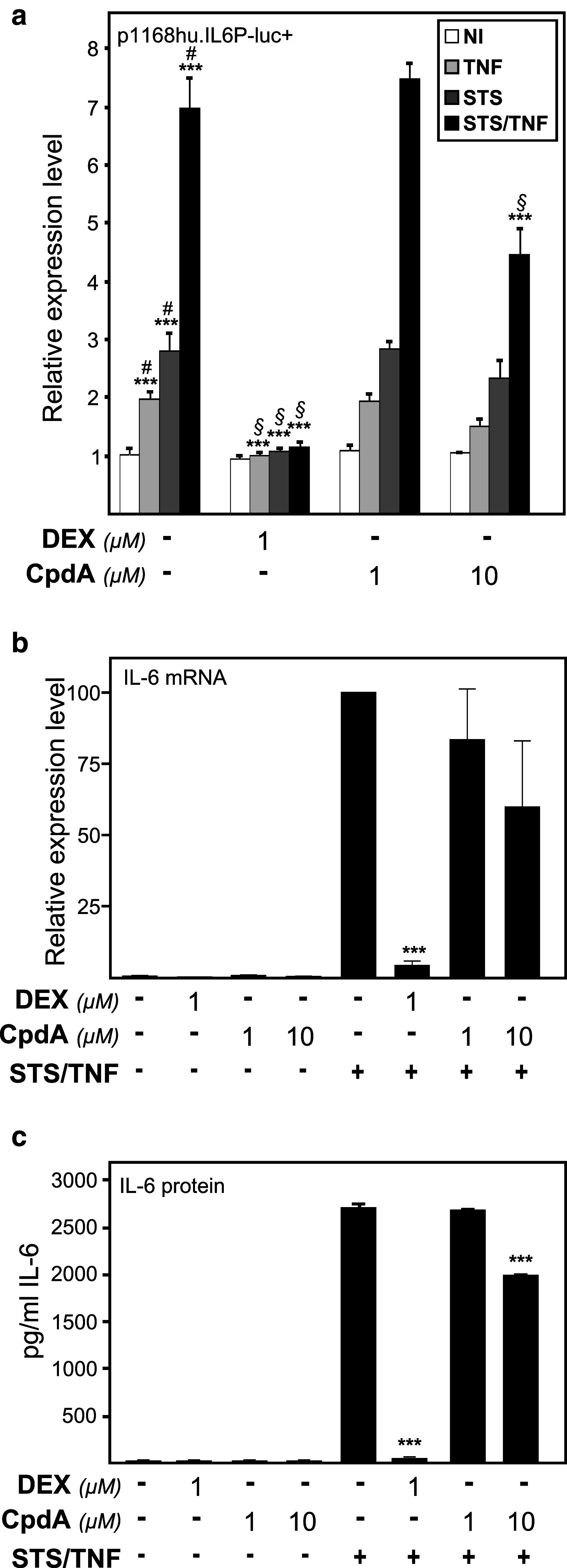
Activation of IL-6 expression parameters by combined NF-κB- and AP-1-activating stimuli is repressed by DEX, but to a lesser extent by CpdA. **a** Subconfluent L929sA cell monolayers, stably integrated with the p1168hu.IL6P-luc+ reporter gene construct, were left untreated or treated with 2,000 IU/ml TNF, with 60 nM STS or a combination hereof, for 5 h, preceded by a 1-h treatment with solvent, DEX (1 μM) or CpdA (1 or 10 μM). At the end of the induction, cell lysates were assayed for reporter gene activities. The experiment was carried out in quadruplicate, and the results are representative of three independent experiments. ***p < 0.001. Comparisons versus the control lane are depicted by ‘#’, comparisons versus the respective pro-inflammatory stimuli are depicted by ‘§’. **b** L929sA cells, starved for 48 h in DMEM devoid of serum, were pre-incubated with solvent, DEX (1 μM), or CpdA (1 or 10 μM) for 1 h, before STS (60 nM) and TNF (2,000 IU/ml) were added, where indicated, for 6 h. Total RNA was isolated and subjected to RT-qPCR assaying IL-6 and two household gene mRNA levels. Specific signal for cDNA of IL-6 was normalized to the averaged household genes signal. The STS/TNF condition was set as 100 and all other conditions were recalculated accordingly to allow ratio comparisons. Total solvent concentration was kept similar in all conditions. Results are shown ± SD. Comparisons were made vs. STS/TNF. The experiment was carried out in triplicate, and the result are averages of two independent experiments. **c** L929sA cells were induced as in **b**. Medium was collected to perform a murine IL-6 ELISA. Protein levels are presented as pg per ml. Total solvent concentration was kept similar in all conditions. Results are shown ± SD. ***p < 0.001. Comparisons were made vs. STS/TNF

A full IL-6 gene promoter activity results from a concerted cooperation between AP-1, CREB, C/EBP, and NF-κB transcription factors [12]. NF-κB has previously been described as a key transcription factor driving tumor necrosis factor (TNF)-induced IL-6 promoter activity [12]. Figure 1a shows the regulation by TNF in the absence or presence of dexamethasone (DEX) or the dissociated GR modulator Compound A (CpdA) of the wild-type human IL-6 gene promoter (a 1,168-bp fragment upstream of the transcription start site), stably transfected in L929sA murine fibroblast cells. As expected, DEX efficiently blocks TNF-induced IL-6 gene expression (Fig. 1a). In contrast, CpdA only marginally represses the TNF-induced IL-6 promoter activity in L929sA cells (Fig. 1a). This modest inhibition is consistently observed (see also Fig. 2a). Upon mutating the AP-1 element, which is present within 300 bp preceding the transcription start site in the IL-6 promoter, both the TNF inducibility of the promoter, and the extent of promoter inhibition by DEX remain similar as for the wild-type construct (Fig. 1b). However, efficient repression is now also reached with CpdA (10 μM), comparable to the repression level that is obtained using DEX (Fig. 1b). This result suggests an inability of CpdA-activated GR to target the AP-1 element of the TNF-induced wild-type IL-6 promoter.

The inhibitory effect of activated GR on NF-κB is a well-known phenomenon [49]. To additionally verify whether CpdA targets NF-κB to the same extent as DEX, we tested their ability to repress a TNF-induced solely NF-κB-driven recombinant promoter construct, carrying a minimal IL-6 promoter-derived TATA box coupled to luciferase. From Fig. 1c, and in contrast to the wild-type IL-6 promoter, it is clear that both CpdA (10 μM) and DEX (1 μM) efficiently block the TNF-induced NF-κB-driven promoter construct (IL6κB)_3_50hu.IL6P-luc+. These results suggest that CpdA-bound GR does not target AP-1 for transrepression in fibroblast cells.

### A strong AP-1-activating signal driving the IL-6 promoter does not concur with an efficient transrepression by CpdA-activated GR

It has been shown before that high inducibility of the IL-6 promoter by the protein kinase inhibitor staurosporine (STS) involves the AP-1, CREB, and C/EBP class of transcription factors [12]. Interestingly, an inducible IL-6 gene expression by mediation of either NF-κB or AP-1 can be distinguished by activation of separate signaling pathways, which can synergize when stimulated simultaneously. Synergistic stimulation of the IL6-promoter has previously been demonstrated upon combined treatment of STS and TNF [12]. In accordance with our earlier results [36], DEX efficiently blocks synergistic IL6 promoter stimulation by NF-κB and AP-1 (Fig. 2a). In sharp contrast, CpdA fails to mediate an efficient transrepression response (Fig. 2a). Combined STS and TNF-induced IL-6 mRNA levels were only marginally affected by CpdA, but efficiently downregulated in the presence of DEX (Fig. 2b). By additionally measuring the corresponding endogenous IL-6 protein levels via ELISA (Fig. 2c), we further established that the regulation of IL-6 protein expression reflects the transcriptional regulation of the IL-6 gene promoter in terms of its inducibility, and also its transrepression capability, using DEX or CpdA (Fig. 2b, c). Whereas DEX is able to inhibit a massive IL-6 production, which was instigated by a combined treatment with STS and TNF, to near baseline levels, CpdA (10 μM) only moderately inhibits this IL-6 protein production (Fig. 2c). Similar results were obtained when cells are stimulated with STS and TNF for 24 h (data not shown). By performing a dose response experiment using L929sA cells with a stably integrated (IL6κB)_3_50hu.IL6P-luc+ plasmid, we verified that the highest dose of CpdA inhibited the combined STS and TNF-induced NF-κB-dependent reporter gene activity (Online Resource 1) equally well as the TNF-induced NF-κB-dependent reporter gene activity (Fig. 1c), i.e., near baseline levels. This result suggests that the selectivity of CpdA to transrepress NF-κB is largely independent of the nature of the NF-κB-activating stimuli.

### In contrast to DEX, CpdA does not transrepress AP-1-driven promoter activity

**Fig. 3:**
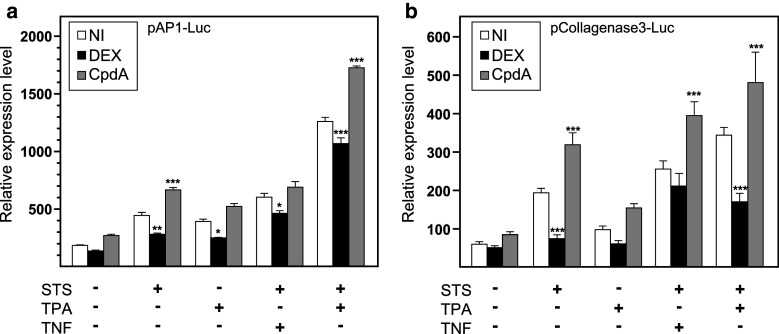
AP-1-driven gene transcription is downregulated by DEX but not by CpdA. **a**, **b** Subconfluent L929sA cell transiently transfected with pAP-1-Luc or pCollagenase-3-Luc were untreated or treated with 2,000 IU/ml TNF, with 60 nM STS, with 50 ng/ml TPA or a combination hereof, for 5 h, preceded by a 1-h treatment with solvent, DEX (1 μM) or CpdA (1 or 10 μM). At the end of the induction, cell lysates were assayed for reporter gene activities. The experiment was carried out in triplicate, and the results are representative of at least two independent experiments. Total solvent concentration was kept similar in all conditions. Results are shown ± SD. *p < 0.05, **p < 0.01, ***p < 0.001. Comparisons were made vs. the respective pro-inflammatory stimuli

The above results prompted us to take a closer look at additional AP-1 repression models with CpdA. To this purpose, we used a recombinant AP-1-driven reporter gene construct, pAP-1-Luc, transiently transfected in L929sA. Additionally, to show that the divergent regulation by CpdA and DEX was unrelated to the nature of the NF-κB and AP-1 stimuli, we changed stimuli into TPA treatment and combinations of STS with TNF or STS with TPA. A combined treatment of STS and TPA results in a synergistic response (Fig. 3a). In contrast to DEX, CpdA failed to transrepress STS-induced, TPA-induced, combined STS and TNF- or STS and TPA-induced AP-1 promoter activities (Fig. 3a). The AP-1-driven *MMP13* gene, coding for the enzyme collagenase3, has previously been described to be susceptible to GC repression [28]. Although this *MMP13* gene is regulated by a more complex AP-1-driven promoter, a similar regulation as for the minimal AP-1-driven promoter is still apparent when assaying pCollagenase3-Luc (Fig. 3b). Interestingly, CpdA did not only fail to transrepress AP-1 driven promoter activity, but seemed actually to enhance the STS and STS/TPA-induced AP-1-dependent promoter activities (Fig. 3a, b). A stably integrated promoter variant of pAP-1-Luc in L929sA cells yielded overall similar results in terms of a refractory transrepression with CpdA (Online Resource 2). It is clear that the divergent transrepression characteristics of DEX and CpdA are largely maintained across different AP-1-reporter gene models, regardless of the AP-1-activating stimuli. As a positive control for the functionality of CpdA, we assayed in parallel the effect of CpdA on a TNF-driven (IL6κB)_3_50hu.IL6P-luc+ promoter construct, stably integrated in L929sA cells. For each experiment, this yielded results that were similar to those presented in Fig. 1c.

### CpdA, in contrast to DEX, sustains AP-1-induced gene and protein expression

**Fig. 4:**
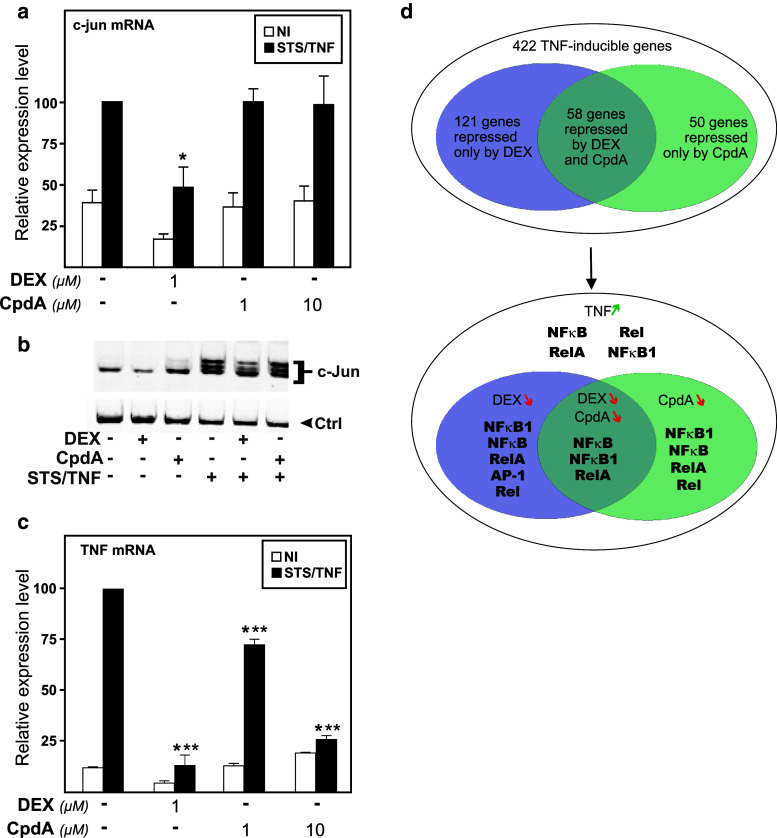
Predominantly AP-1-regulated target genes are only transrepressed by DEX- and not by CpdA-activated GR, but promoter complexity determines the final outcome. **a**, **c** L929sA cells, starved for 48 h in DMEM devoid of serum, were pre-incubated with solvent, DEX (1 μM), or CpdA (1 or 10 μM) for 1 h, before STS (60 nM) and TNF (2,000 IU/ml) were added, where indicated, for 6 h. Total RNA was isolated and subjected to RT-qPCR assaying cellular *c*-*jun*, TNFα, and β-actin and hypoxanthine–guanine phosphoribosyltransferase (HPRT) household gene mRNA levels. Specific signal for cDNA of *c*-*jun* or *TNFα* was normalized to the averaged household genes signal. The STS/TNF condition was set at 100 and all other conditions were recalculated accordingly to allow ratio comparisons. Total solvent concentration was kept similar in all conditions. Results are shown ± SD. The experiment was carried out at least in triplicate and the results are averages of at least two independent experiments. Results of the statistical analysis via ANOVA followed by a Tukey multiple comparison post-test are shown for particular groups of interest, in comparison to the STS/TNF group). **b** L929sA cells, starved for 48 h in DMEM devoid of serum, were treated with solvent, a combination of STS (60 nM) and TNF (2,000 IU/ml) for 5 h in absence or presence of a 1 h pretreatment of DEX (1 μM) or CpdA (10 μM). Total protein extracts were prepared in duplicate and subjected to Western-blot analysis to detect c-Jun protein. Detection of NF-κB p65 served as a loading control (Ctrl). The result is a representative of three independently performed experiments. **d** A549 cells, starved for 24 h in DMEM devoid of serum, were pretreated for 1 h either with solvent, DEX (1 μM) or CpdA (10 μM), either or not followed by a 3-h treatment with TNF (2,000 IU/ml). Gene expression levels of corresponding RNA samples were evaluated by a whole genome transcriptome Agilent array (*upper panel*). Genes with adjusted *p* values lower than 0.05 in at least one contrast and a fold change higher than 1.3 were selected as significant. Pscan analysis with a minimal statistical significance of *p* < 0.05, indicates enrichment for specific transcription factor binding motifs in the corresponding gene promoters. Here, we mention the identified NF-κB and AP-1 family members in *bold* (*lower panel*)

To find out how CpdA may affect the transcriptional regulation of an endogenous AP-1-controlled gene, we next analyzed its effect on *c*-*jun* mRNA levels via qPCR analysis. Only DEX, but not CpdA, ensured an efficient transrepression of STS/TNF-induced *c*-*jun* mRNA levels (Fig. 4a) and a concomitant repression of c-Jun protein levels (Fig. 4b). At the c-Jun protein level, and opposite to DEX, we also noted a slight upregulatory effect with CpdA alone (Fig. 4b). These results point to distinct effects of DEX and CpdA in regulating AP-1-mediated protein production. As a positive control for the functionality of CpdA and to further explore potential differences in target gene regulations, we studied in parallel the mRNA regulation of a number of other genes with a role in inflammation, i.e., the TNFα gene, the chemokine gene MCP-1, and the anti-inflammatory gene coding for IκBα. From Fig. 4c it can be concluded that both CpdA (10 μM) and DEX (1 μM) are able to efficiently transrepress the STS/TNF-induced levels of TNFα mRNA. A similar regulation could be noted for MCP-1 (Online Resource 3a).

The mRNA regulation of the anti-inflammatory IκBα gene (*NFκBia*) shows a good inducibility upon treatment with STS and TNF and is a target for transrepression by CpdA, but not DEX (Online Resource 3b). In line with the presence of a GR-responsive binding motif in the proximal promoter region of the IκBα gene [50], DEX alone was able to enhance basal levels of IκBα mRNA (Online Resource 3b). As reported before [34], the combination of a pro-inflammatory stimulus with DEX does not further enhance mRNA expression levels of IκBα (Online Resource 3b). This result is most likely the net effect of a stimulatory signal on the GRE element, combined with an inhibitory signal on the NF-κB element. Finally, the dissociated character of CpdA-activated GR is confirmed by measuring mRNA levels of the standard GRE-driven GILZ gene, which is only elevated in the presence of DEX (Online Resource 3c).

To generalize the validity of our findings, we broadened our gene analysis and meanwhile expanded to an alternative cell system and species. In that respect, we assayed human A549 lung epithelial cells via an Agilent expression analysis. Results show that of 422 TNF-inducible genes, 179 genes were repressed by DEX. From this gene pool, 58 genes were in turn also repressed by CpdA and 121 genes appeared to be exclusively targeted by DEX (Fig. 4d; Online Resource 4). Exemplary gene expression profiles of either group are displayed in Online Resource 5). Analysis via the bioinformatics tool Pscan [44] of the TNF-stimulated and DEX and/or CpdA–repressed genes (Online Resource 4) with a focus on NF-κB and AP-1 family members, furthermore showed a possible discrimination in promoter motifs between DEX and CpdA-targeted gene promoters, as gene promoters which contain a NF-κB family member-recognizing motif can be induced by TNF and repressed by both DEX and CpdA, whereas the additional presence of an AP-1 transcription factor binding motif allows DEX repression, but not CpdA repression.

**Fig. 5:**
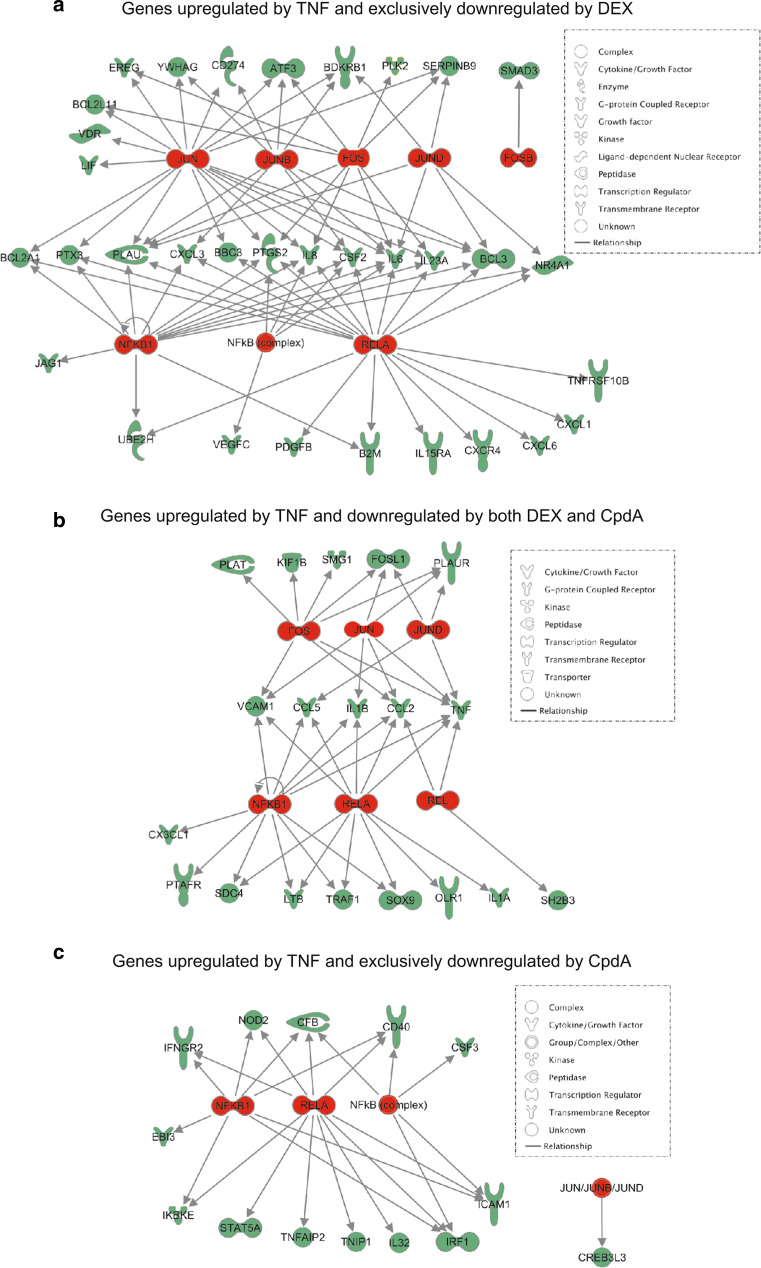
Graphical illustration of differentially regulated AP-1 and NF-κB targets in the dataset. *Arrows* indicate experimentally confirmed promoter binding or regulation of expression. Transcription factors are depicted in* red* and their respective target genes are displayed in *green*. Only subunits with at least one unique target were kept to avoid redundancy. Promoter analysis of the six targets with no known dependency on NF-κB (**b** and **c**) revealed the presence of NF-κB motifs, indicating possible contribution of this transcription factor to their transcriptional regulation (2000–2013 Ingenuity Systems, Inc. All rights reserved)

To further support the Pscan analysis for enrichment of specific transcription factor-binding motifs, we performed an Ingenuity pathway analysis (IPA) on the TNF-upregulated and DEX- and/or CpdA-downregulated gene sets, as listed in Online Resource 4, thus capturing the essence of the transcription factors regulated by DEX and CpdA. This analysis focuses on the enrichment of known NF-κB- and AP-1-regulated target genes in the dataset and shows that both NF-κB and AP-1 targets are highly overrepresented among TNF-stimulated genes that are repressed solely by DEX (Table 1; Fig. 5a). In contrast, TNF-stimulated genes that are repressed by both DEX and CpdA or solely CpdA, show a relatively higher enrichment of NF-κB target genes as compared to AP-1 target genes (Table 1; Fig. 5b–c). Indeed, even though SMG1, KIF1B PLAT, FOSL1, PLAUR and CREB3L3, are not identified as NF-κB-dependent target genes via IPA analysis in Fig. 5b, c, all of these gene promoters are marked by the presence of a binding motif for a NF-κB family member in their proximal promoter (Table 2).

**Table 1:** Enrichment of NF-κB and AP-1 target genes in A549 cells in the dataset as identified by IPA

Upstream regulator	*p* value of overlap	Target molecules in dataset
*Genes upregulated by TNF and exclusively downregulated by DEX*		
RELA	1.23E−17	B2M,BBC3,BCL2A1,BCL3,CSF2,CXCL1,CXCL3,CXCL6,CXCR4,IL15RA,IL23A,IL6,IL8, JUN,NFKB1,NR4A1,PDGFB,PLAU,PTGS2,PTX3,TNFRSF10B,UBE2H
JUN	8.35E−13	ATF3,BBC3,BCL2A1,BCL2L11,BCL3,CD274,CSF2,EREG,IL23A,IL6,IL8,JUN,LIF,PLAU, PTGS2,PTX3,SERPINB9,VDR,YWHAG
NFκB1	1.54E−12	B2M,BCL2A1,BCL3,CSF2,CXCL3,IL23A,IL6,IL8,JAG1,NFKB1,NR4A1,PLAU,PTGS2,PTX3, UBE2H
REL	4.23E−12	B2M,BBC3,BCL2A1,BCL3,CSF2,IL23A,IL6,IL8,JUN,NFKB1,NR4A1,TNFRSF10B
JUNB	1.26E−07	ATF3,BCL3,CD274,CSF2,CXCL3,IL6,PLAU,YWHAG
NFκB (complex)	2.08E−05	CSF2,IL6,IL8,NFKB1,PTGS2,VEGFC
JUND	2.58E−05	BCL3,IL6,NR4A1,PLAU,SERPINB9
FOS	3.44E−05	ATF3,BCL2L11,EREG,FOSB,IL23A,IL6,IL8,JUN,PLAU,PLK2,PTGS2,SERPINB9
*Genes upregulated by TNF and downregulated by both DEX and CpdA*		
NFκB1	4.95E−13	CCL2,CCL5,CX3CL1,IL1B,LTB,NFKB2,PTAFR,SDC4,SOX9,TNF,TRAF1,VCAM1
RELA	6.72E−11	CCL2,CCL5,IL1A,IL1B,LTB,NFKB2,OLR1,SDC4,SOX9,TNF,TRAF1,VCAM1
JUND	2.34E−05	CCL5,FOSL1,PLAUR,TNF
FOS	6.81E−05	CCL2,FOSL1,KIF1B,PLAT,PLAUR,SMG1,TNF,VCAM1
REL	3.27E−04	CCL2,NFKB2,SH2B3,TNF
JUN	6.69E−04	CCL2,FOSL1,IL1B,PLAUR,TNF,VCAM1
*Genes upregulated by TNF and exclusively downregulated by CpdA*		
RELA	7.50E−12	CD40,CFB,ICAM1,IFNGR2,IKBKE,IL32,IRF1,NOD2,RELA,STAT5A,TNFAIP2,TNIP1
NFκB1	1.31E−09	CD40,CFB,EBI3,ICAM1,IFNGR2,IKBKE,IRF1,NOD2,RELA
NFκB (complex)	3.01E−06	CD40,CFB,CSF3,ICAM1,IRF1
JUN/JUNB/JUND	1.93E−02	CREB3L3

*p* values indicate the significance of overlap (Fisher’s exact test) between genes in the dataset and targets of the respective transcription factor

**Table 2:** Presence of NF-κB family member motifs in promoters of FOS/JUN target genes (SMG, KIF1B, PLAT, FOSL1, PLAUR, CREB3L3), as identified via Pscan

Symbol	Score	Position	Sequence	Strand
SMG1	0.91	−239	GGGGATCTCCA	–
KIF1B	0.88	−432	GGGGGTCACCC	+
PLAT	0.86	−435	GGGGCACCTCC	+
FOSL1	0.84	−154	GGGGCTCCACC	+
PLAUR	0.81	−432	GGGGTTTCACC	+
CREB3L3	0.82	−201	GGGGTACCTCC	–

Pscan analysis was run with the following setting: Organism: *H. Sapiens*; Scan region: −450/+50 b: Motif Database: Jaspar. The table shows a summary for hits of the NFκB1 motif (MA0105.1)

Combining these data, we conclude that in line with our hypothesis, all genes that are regulated by both DEX and CpdA, have been either previously identified as NF-κB targets or have a NF-κB family member binding site in their promoter (Tables 1, 2; Figs. 4d, 5).

### CpdA blocks ERK activation but sustains JNK activation in L929sA

**Fig. 6:**
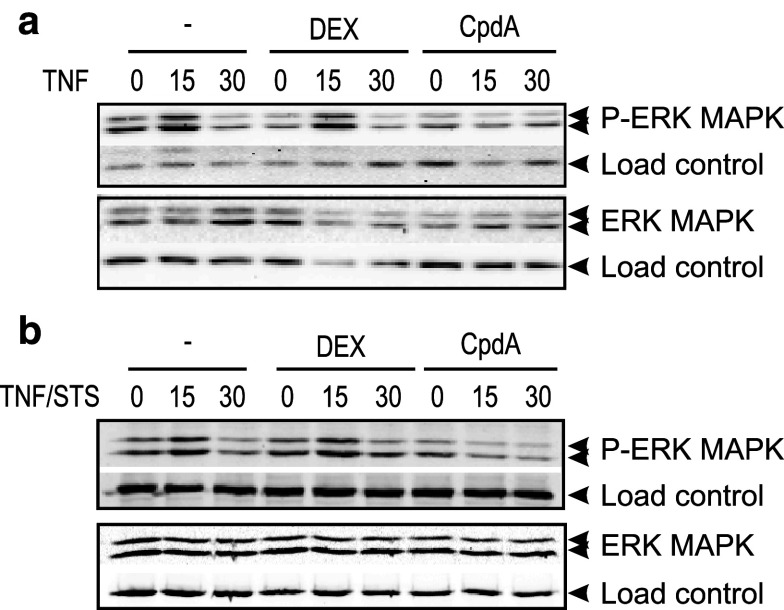
CpdA, but not DEX, blocks activated ERK in fibroblasts. **a**, **b** L929sA cells, starved for 48 h in DMEM devoid of serum, were pretreated with solvent, 1 μM DEX, or 10 μM CpdA for 1 h, followed by either or not TNF (2,000 IU/ml) (**a**), or TNF combined with STS (60 nM) (**b**), for the indicated time points (in minutes). Cell lysates were made and activated ERK was detected using the phospho-specific ERK MAPK antibody. Aspecific bands, non-phosphorylated proteins, and/or NF-κB p65 served as a loading control (*indicated as load control*)

The observation that a differential AP-1 regulation by DEX and CpdA may fine-tune GR transrepression efficacies is intriguing. In an attempt to find a plausible explanation for this phenomenon, we studied DEX- and CpdA-mediated regulation of MAPK activation patterns. In analogy with previous findings [36], DEX does not block TNF-activated phospho-ERK MAPK in L929sA (Fig. 6a), and similar time-kinetics and results were noted for STS/TNF-activated phospho-ERK MAPK (Fig. 6b), suggesting that a different MAPK-activating signal does not lead to a different regulation by GCs. In contrast to DEX, CpdA impedes both the TNF and STS/TNF-induced phosphorylation of ERK MAPK (Fig. 6a–b).

**Fig. 7:**
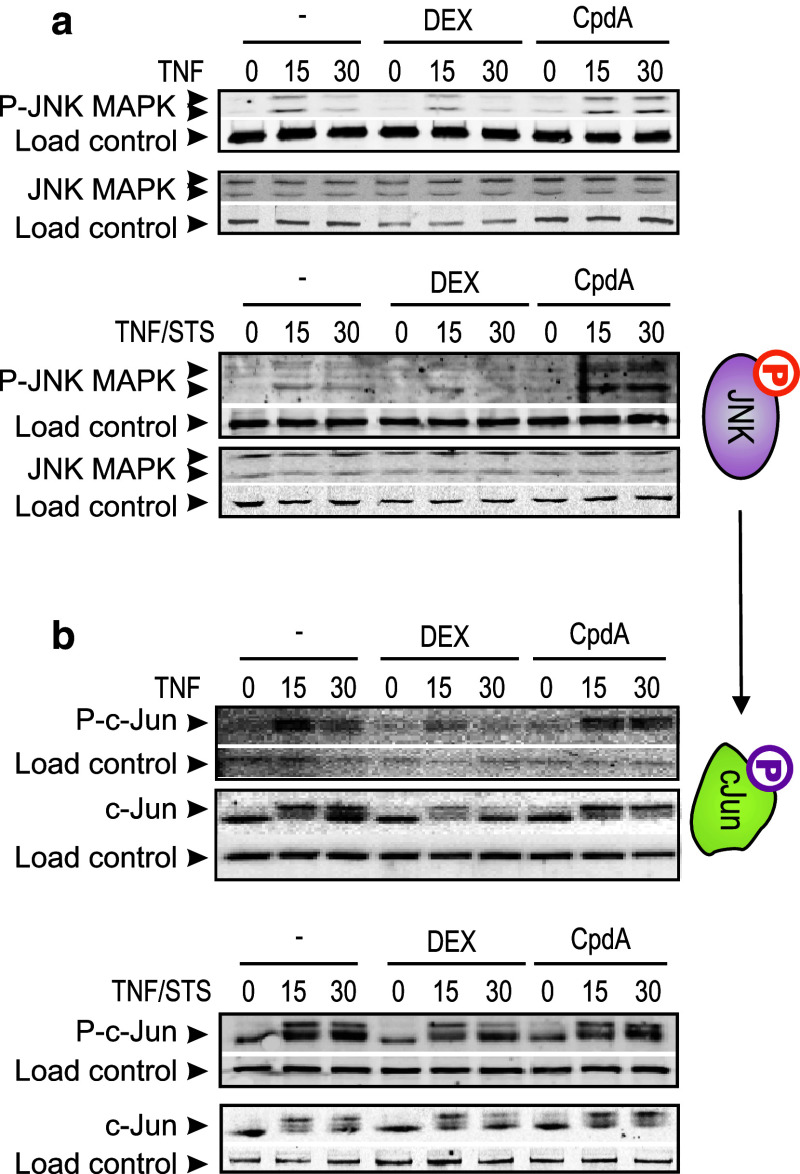
CpdA sustains activated JNK and c-Jun in fibroblasts. **a**, **b** L929sA cells, starved for 48 h in DMEM devoid of serum, were pretreated with solvent, 1 μM DEX, or 10 μM CpdA for 1 h, followed by either or not TNF (2,000 IU/ml) or TNF combined with STS (60 nM) for the indicated time points (in minutes). Cell lysates were made and activated JNK (**a**) was detected using the phospho-specific JNK MAPK antibody and activated c-Jun (**b**) was detected using the phospho-specific c-Jun antibody. Aspecific bands, non-phosphorylated proteins and/or NF-κB p65 served as a loading control (*indicated as load control*)

JNK activation in L929sA cells was found to be very weak, using either TNF or STS/TNF. However, CpdA prolongs both TNF and STS/TNF-induced activation of JNK beyond the 15-min time point (Fig. 7a). Of note, the persistent phosphorylation of JNK MAPK in presence of CpdA, is reflected in a similar phosphorylation profile of the downstream JNK target, c-Jun (Fig. 7b) and contrasts with the regulation by DEX. Finally, neither DEX nor CpdA can differentially modulate the p38 MAPK phosphorylation profiles (Online Resource 6). Altogether, the observed differential effects on c-Jun phosphorylation may explain why classic AP-1-driven gene expression remains unaffected or could be even slightly enhanced by CpdA-loaded GR, but is on the other hand readily repressed by DEX-activated GR (Figs. 3, 4a; Online Resource 4 and 5).

### GR is essential to mediate the gene expression modulation effect of DEX and CpdA

**Fig. 8:**
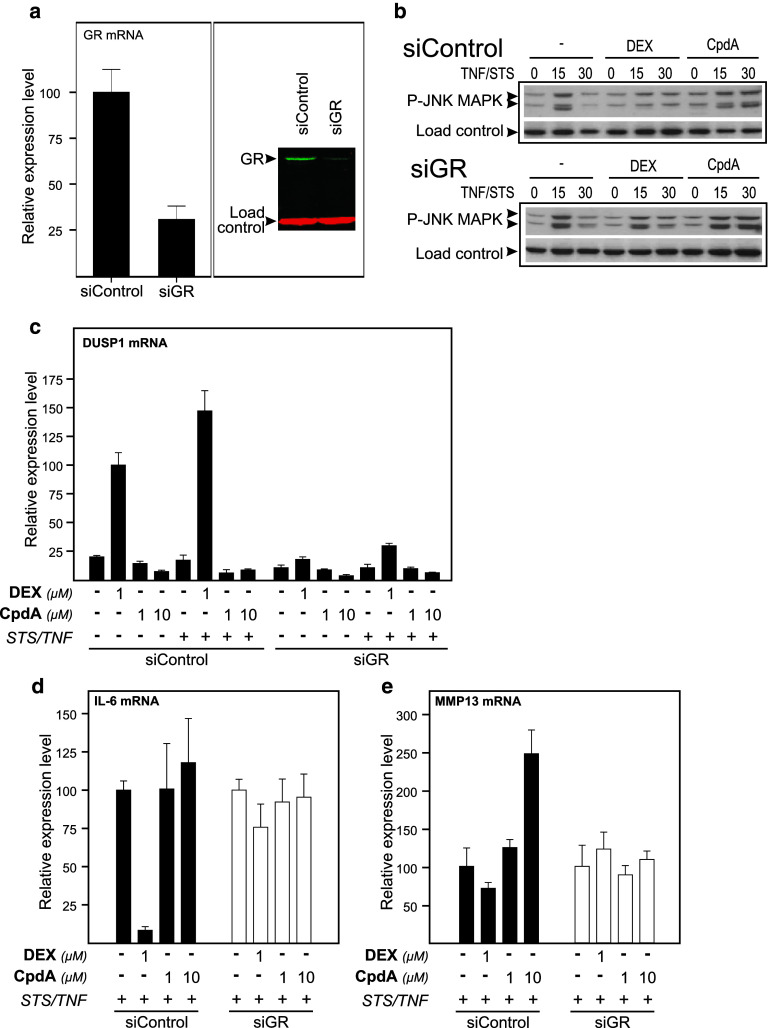
GR is essential to mediate the gene expression modulation effect of DEX and CpdA. L929sA cells were transfected with siRNA control (siControl) or siRNA targeted at GR (siGR) and were allowed to rest for 48 h post transfection. In the 16-h period before induction or sampling, cells were starved in DMEM devoid of serum. **a** We controlled for the efficiency of siRNA GR targeting. Total RNA was isolated and subjected to RT-qPCR for GR mRNA levels and expression levels were normalized to housekeeping gene controls. The expression levels for GR (siControl) were set at 100 and the siGR condition was recalculated accordingly (*left panel*). Cell lysates were made and GR protein was visualized via Western-blot analysis (*right panel*). Actin served as a loading control. **b** SiRNA-transfected L929sA cells (siControl or siGR) were pretreated with solvent, 1 μM DEX, or 10 μM CpdA for 1 h, followed by either or not TNF (2,000 IU/ml) combined with STS (60 nM), for the indicated time points (in minutes). Cell lysates were made and activated JNK was detected using the phospho-specific JNK MAPK antibody. NF-κB p65 served as a loading control (*indicated as load control*). **c**–**e** SiRNA-transfected L929sA cells (siControl or siGR) were pre-incubated with solvent, DEX (1 μM), or CpdA (1 or 10 μM) for 1 h, before TNF (2,000 IU/ml) and STS (60 nM) was added, where indicated, for 6 h. Total RNA was isolated and subjected to RT-qPCR for specific target genes, and expression levels in each treatment group were normalized to housekeeping gene controls. Normalized mRNA levels for DUSP1 (**c**) was presented with the DEX (SiControl) set as 100 and all other conditions were recalculated accordingly. Normalized mRNA levels for IL-6 (**d**) and MMP13 (**e**) were presented with the STS/TNF condition (siControl and siGR) set at 100 and all other conditions were recalculated accordingly to allow ratio comparisons. Total solvent concentration was kept similar in all conditions. Results are shown ± SD. The qPCR was carried out at least in triplicate. Results are representative for two independent experiments

To investigate whether our observations occur in a GR-dependent or -independent manner, we used siRNA in L929sA fibroblasts. Controls via assaying GR mRNA levels via qPCR and GR protein levels via Western blot (Fig. 8a) revealed an efficient GR knock down of nearly 75 %. Firstly, we analyzed whether the presence of GR is pivotal to the effect of Compound A on JNK phosphorylation. Knockdown of GR in L929sA cells shows that a diminishment in GR protein can partially revert the negative impact of DEX on STS- and TNF-stimulated JNK phosphorylation, suggesting a GR dependence. However, a decline in GR protein levels does not seem to alter the positive effects of Compound A on sustaining this STS- and TNF-stimulated JNK phosphorylation (Fig. 8b), arguing for a possibly GR-independent regulation. As JNK phosphorylation is targeted by the DUSP1 phosphatase [51], we wondered how CpdA would effect the production of DUSP1 mRNA. As expected, CpdA does not stimulate the activation of the GRE-regulated DUSP1 gene promoter, while DEX in a GR-dependent manner does so (Fig. 8c). Hence, the apparent absence of GR dependence in CpdA-mediated regulation of JNK phosphorylation might be explained by the lack of DUSP1 synthesis.

Upon investigating the STS- and TNF-stimulated IL-6 gene expression, DEX-mediated repression of IL-6 can be reverted by knockdown of GR in both L929sA and A549 cells (Fig. 8d Online Resource 7b). In CpdA-treated cells, this GR knockdown has only a limited effect on what is already a marginal repression profile in A549 cells, when compared to DEX (Online Resource 7b-c). However, a knockdown of GR resulted in a clear ablation of the stimulatory effect of CpdA on STS/TNF-regulated MMP13 gene expression in L929sA cells (Fig. 8e) and *c*-*Jun* gene expression in A549 cells (Online Resource 7d), indicating that for AP-1 regulated genes GR is necessary to mediate the CpdA-induced elevation of the STS and TNF-stimulated gene expression. To summarize, the presence of GR is essential to mediate the gene regulatory effect of DEX and CpdA, but appears to be redundant for the CpdA-mediated prolongation of JNK phosphorylation.

### CpdA, in contrast to DEX, does not support GR recruitment onto the AP-1-dependent *c*-*jun* gene promoter

**Fig. 9:**
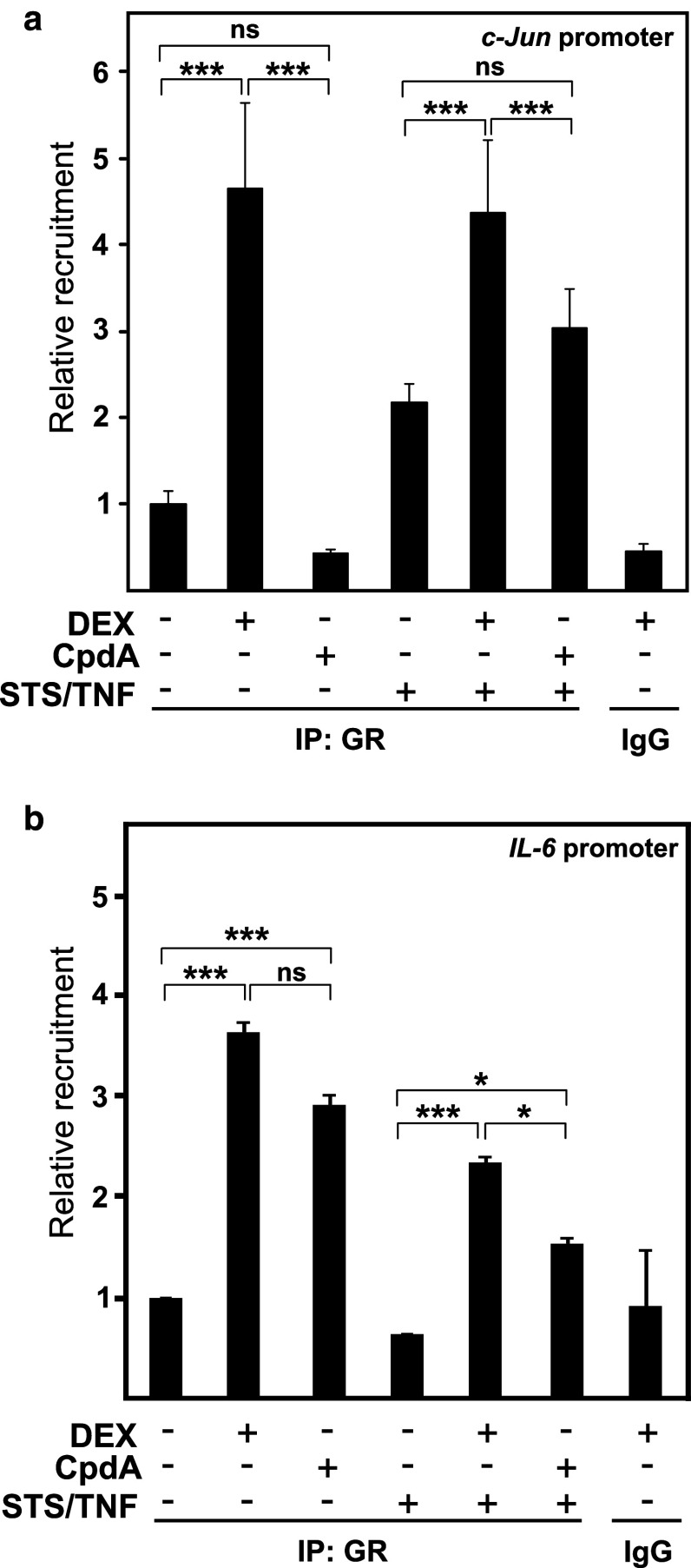
Only DEX recruits activated GR to the *c*-*jun* gene promoter. **a**, **b** L929sA cells, serum-starved for 48 h in DMEM devoid of serum, were pretreated for 1 h with solvent, DEX (1 μM), or CpdA (10 μM). Ensuing the indicated stimulation with TNF (2,000 IU/ml) combined with STS (60 nM) for 30 min, cells were lysed and total cell extracts were subjected to ChIP analysis and subsequent qPCR, detecting GR protein recruitment at the *c*-*jun* or IL-6 gene promoters. qPCR signal of immunoprecipitated *c*-*jun* or IL-6 promoter fragments is presented relative to input data. Averaged results of at least two independent experiments are shown ± SD. *p < 0.05; **p < 0.01, and ***p < 0.001

The failure of CpdA to block JNK MAPK activation, in the presence of TNF or its combination with STS, may explain CpdA’s deficiency to inhibit AP-1-mediated transcription. To define whether CpdA-activated GR is still recruited onto AP-1-driven gene promoters under those conditions, we performed a chromatin immunoprecipitation (ChIP) analysis for endogenous GR in L929sA cells, using a primer set proximal to the AP-1-binding sites in the *c*-*jun* gene promoter. Figure 9a shows that DEX can enhance GR promoter occupancy at the above-mentioned *c*-*jun* gene promoter region. However, CpdA-activated GR does not show a statistically significant elevated recruitment at this gene promoter. When studying the recruitment of GR near the NF-κB-binding site of the IL-6 promoter (Fig. 9b), we observe that in absence of the pro-inflammatory stimulus STS/TNF, GR is equally well recruited onto the basal IL-6 promoter in presence of DEX (1 μM) or CpdA (10 μM). However, following AP-1 stimulation with STS/TNF, a more pronounced GR recruitment can be detected in presence of DEX as compared to CpdA. Nevertheless, the IL-6 promoter does still show an enhanced GR promoter occupancy in the combined presence of CpdA and STS/TNF (Fig. 9b), presumably because of transcription factor region overlaps.

### JNK2 is involved in the CpdA-mediated sensitization to TNF toxicity

**Fig. 10:**
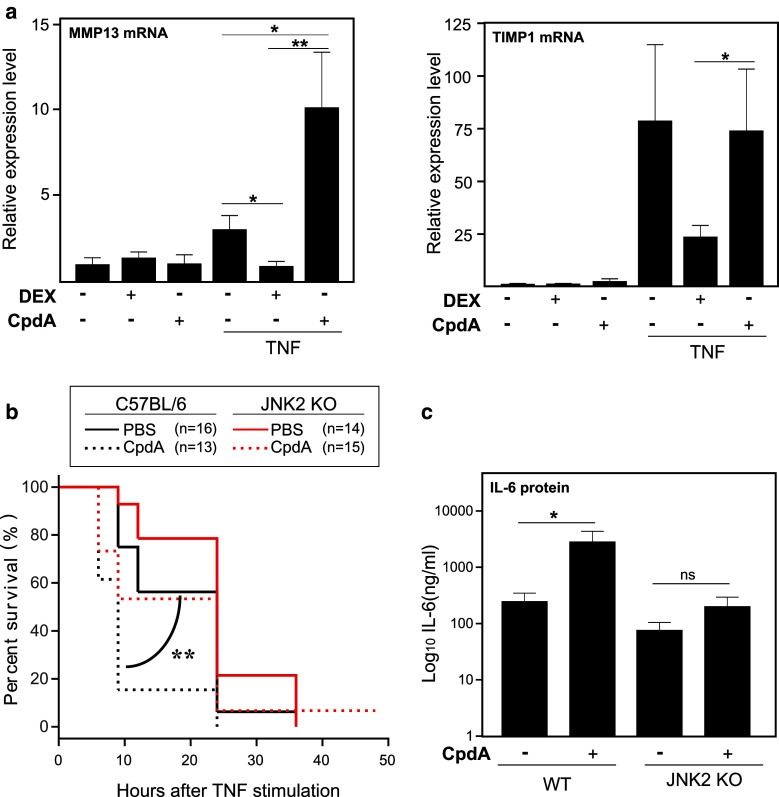
CpdA enhances TNF-induced AP-1-driven gene expression in vivo and JNK2-/- mice are resistant to the CpdA-mediated hypersensitivity to TNF-induced lethality. **a** Wild-type C57BL6/J mice were injected i.p. with solvent or TNF (25 μg) for a total of 6 h, in the presence or absence of DEX (10 mg/kg) or CpdA (8 mg/kg), which was administered 30 min before the solvent (PBS) or TNF administration. Liver mRNA was assayed for levels of *MMP13* and *TIMP1.* **b** Wild-type C57BL6/J mice and JNK2^−/−^ mice were injected i.p. with solvent or TNF (25 μg) in the presence or absence of CpdA (8 mg/kg). Survival of wt (*black lines*, *n* = 13–16) and JNK2^−/−^ mice (*red lines*, *n* = 14–15) was monitored for the indicated time points. **c** Serum IL-6 protein levels were measured in wt and JNK2^−/−^ mice 6 h after the challenge with TNF (25 μg), in the presence of either PBS or CpdA (8 mg/kg). Averages of 2 independent experiments are shown for (**a**) and (**b**). *ns* not significant; *p < 0.05; **p < 0.01

So far, our findings were restricted to cellular models of fibroblasts and epithelial cells. To expand the validity of our conclusions in vivo, we treated C57BL6/J mice with CpdA or DEX for 6 h, in absence or presence of 25 μg of TNF, and subsequently we analyzed liver mRNA levels for AP-1 target genes, *MMP13*, *TIMP1*, (Fig. 10a) and *c*-*jun* (data not shown). We could demonstrate that CpdA treatment, in the presence of TNF, coincides with an increased *MMP13* expression, while for *TIMP1* mRNA expression, CpdA does not affect the TNF-induced transcription. For *c*-*jun*, overall levels were unaffected by TNF, DEX, CpdA, or combinations thereof (data not shown), probably indicating that the killing time point is suboptimal to detect a modulation of this gene.

To explore the functional relevance of our findings, we switched to an animal model of systemic inflammation. Recently, it has been shown that mice expressing a dimerization-defective GR (GR^dim/dim^) exhibit an increased sensitivity towards TNF lethality [30]. GR-mediated control of TNF-induced inflammation involves the induction of MKP-1. Moreover, in MKP1^−/−^ mice, which exhibit a more pronounced sensitivity to TNF and thus TNF-induced lethality, the phosphorylation of JNK was enhanced. Since JNK2^−/−^ mice showed significant protection against TNF-induced lethality, JNK2 was identified as an essential player in in vivo inflammation as induced by TNF.

Since the present data demonstrate that CpdA leads to a sustained JNK/AP-1 activity in fibroblasts, we asked whether CpdA may aggravate TNF-induced lethality in vivo, via JNK2 activation. Hereto, we injected wt mice and JNK2^−/−^ mice i.p. with 25 μg TNF (a lethal dose) followed by CpdA (8 mg/kg), the dose that was previously found to be effective in the mouse model for EAE) and monitored survival. As hypothesized, CpdA sensitizes mice to TNF-mediated lethality, an effect that is counteracted in JNK2^−/−^ mice (Fig. 10b). Since IL-6 levels are a good indicator for TNF sensitivity [52] we assayed IL-6 serum protein levels. In TNF-treated wt mice, CpdA enhanced IL-6 serum levels (Fig. 10c), which correlates with the observed enhanced lethality (Fig. 10b). In contrast, in a JNK2^−/−^ background, IL-6 protein levels remained largely unaffected by the addition of CpdA (Fig. 8c), which again is in concordance with the observed similar extent of survival (Fig. 10b).

## Discussion

**Fig. 11:**
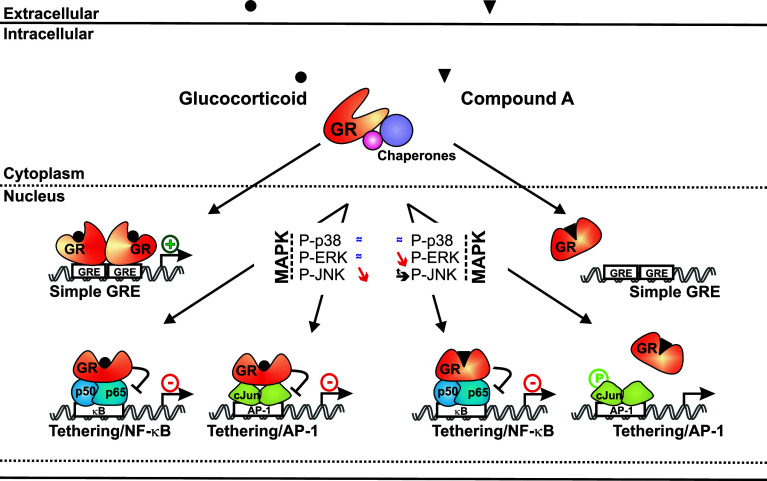
Summarizing model for the transcription factor-selective action of CpdA versus DEX. The cytoplasmic GR is kept in a ligand-receptive conformation by binding to chaperone molecules. Upon binding of either GCs or CpdA, the GR changes its specific conformation and translocates into the nucleus. GC-bound GR can form a homodimer and as such binds to a palindromic simple GRE, thus propagating its classic transactivation mechanism. Conversely, CpdA-bound GR cannot form homodimers and is therefore not able to bind a simple GRE or support transactivation. While GC-bound GR leaves p38 and ERK MAPK phosphorylations (*almost equal to symbol*) unaffected, it diminishes JNK MAPK phosphorylation (*slanting down arrow*). Stimulation with CpdA affects these MAPK phosphorylations differently, as it actually prolongs JNK MAPK phosphorylation (*arrow*) and sparks a decline in ERK MAPK phosphorylation (*slanting down arrow*). Also CpdA does not affect the level of p38 MAPK phosphorylation (*almost equal to symbol*). As expected from the differential MAPK phosphorylation modulations, CpdA- and GC-bound GRs also show a differentiation in transcription factor targeting. GC-bound GR is recruited onto NF-κB- and AP-1-driven gene promoters and is fully capable of transrepressing both NF-κB- and AP-1-driven gene expression. In contrast, CdpA-bound GR can only bind to NF-κB-driven gene promoters and not to AP-1-driven gene promoters. As such, CpdA-bound GR only supports transrepression of NF-κB-mediated gene transcription, and not AP-1-mediated gene transcription

A major challenge in nuclear receptor biology is the identification of ligands with a transcription factor and/or gene-selective action, influencing diseases through the regulation of a subset of target genes and not across their entire gene-regulatory repertoire [53]. NF-κB and AP-1 are known inflammatory mediators and thus well-described transcription factor targets of classic GR-mediated transrepression. In the current work, we present data demonstrating that the GR modulator CpdA, besides its ability to dissociate transrepression from transactivation, is able to also discriminate between repression modes of NF-κB and AP-1, with a pronounced preference for the inhibition of NF-κB. So far, most of the steroidal GR ligands described are capable of transrepressing both NF-κB and AP-1 [7, 54, 55]. We describe here that CpdA, in contrast to classic GCs, does not block but rather sustains AP-1-driven gene expression. The underlying mechanism most probably involves a differential modulation of the JNK kinase and an impaired recruitment of CpdA-activated GR onto AP-1-driven gene promoters. These findings are summarized in a model (Fig. 11).

Upon studying the activated IL-6 promoter, stably integrated in L929sA fibroblast cells, we consistently observed a stronger inhibitory potential of GCs as compared to CpdA (Figs. 1a, 2). Reporter gene analysis results using the 1,168 bp IL-6 promoter element (Fig. 1a) were supported by IL-6 mRNA and protein expression data (Fig. 2b, c), confirming a less efficient transrepression capacity of CpdA as compared to DEX, on IL-6 in fibroblasts. These consistent observations lead us to hypothesize that CpdA may preferentially target only a subset of GR-targeted transcription factors in the IL-6 gene promoter. Three different strategies were followed to corroborate the working hypothesis that CpdA may favor NF-κB repression over AP-1 repression. First, efficient CpdA-mediated GR transrepression is restored in reporter gene experiments with an IL-6 gene promoter variant with a point-mutation in the AP-1 binding site (Fig. 1b). Second, and as reported before [35], the recombinant triple NF-κB-binding element-containing promoter, flanking a 50 bp minimal IL-6 promoter (p(IL6κB)_3_50hu.IL6P-luc+), is equally responsive to the inhibitory potential of CpdA and DEX (Fig. 1c; Online Resource 1). Third, direct proof for impaired AP-1 repression by CpdA, was provided in cellular studies analyzing either recombinant or physiological AP-1-driven promoters (Figs. 3, 4a, b; Online Resource 2, 7d) and was additionally supported by transcriptomic studies (Figs. 4d, 5; Table 1; Online Resource 4 and 5) and by analyzing AP-1 target gene expression using liver tissue from mice injected with CpdA and TNF (Fig. 10a). Promoters regulated by AP-1 elements, but not NF-κB elements proximal to the transcription start site, include those ensuring the expression of *c*-*jun* and matrix metalloproteinase members (MMPs), including *MMP13 (collagenase*-*3)*. Recombinant AP-1 regulated promoters, either transiently or stably transfected into L929sA cells and regardless of the activating stimuli, are not repressed by CpdA-bound GR, while their activity is efficiently inhibited by classic GC-activated GR (Fig. 3; Online Resource 2). In accordance with the reporter gene assays, only DEX but not CpdA repressed the synergistic activation of *c*-*jun* mRNA levels (Fig. 4a), which is further corroborated by protein expression data for c-Jun (Fig. 4b). Similar results were obtained via a microarray analysis of an alternative cell line, namely A549 lung epithelial cells (Online Resource 5a, 7), arguing against a cell type-specific effect. In vivo results even demonstrate a significant CpdA-mediated enhancement of the TNF-induced *MMP13* gene expression in liver, corresponding to in vitro results in L929sA cells (Fig. 8e). Applying software for transcription factor binding site prediction [44] revealed a long list of well-known cytokine and chemokine promoters carrying a NF-κB- as well as an AP-1-responsive element (or multiple ones) in their promoter regions within 450 bps of the transcription start site. Examples hereof are the interleukins (IL-6, IL-8, IL-1β), TNF, MCP-1, COX-2, E-selectin, and A20. A Pscan analysis of A549 transcriptomics suggests an overall possibility to discriminate between CpdA and/or DEX responsiveness on the basis of featured transcription factor binding sites, in which the presence of AP-1- binding motifs seems to be an indication of an additional responsiveness to DEX, as compared to CpdA (Fig. 4d). IPA analysis zeroing in on NF-κB and AP-1-regulated target genes confirms the ligand-induced discrimination between these two transcription factors (Tables 1, 2; Fig. 5).

The question arises as to whether the transcription factor-specific effects we report here for transrepression by CpdA versus DEX in fibroblast cells are a rigid and general phenomenon. Gene promoter-specific effects (e.g., the identity of flanking residues, or the distance between regulatory elements thus influencing promoter looping effects) may probably play an additional and decisive role, since we observed that some cytokines that are indeed co-regulated by NF-κB and AP-1, still demonstrate an efficient transrepression by CpdA. Hence, caution is warranted, as the presence of a potential AP-1 binding site does not always allow to predict insensitivity towards CpdA-mediated repression. Exemplary, the TNF gene itself was efficiently repressed by CpdA (Fig. 4c, Online Resource 4), as was the MCP-1 gene (Online Resource 3a). Similarly, in another study it was found that the TNF-induced *MMP1* gene expression was as efficiently downregulated by DEX as by CpdA in synovial fibroblasts [56]. Conceivably, CpdA effects on other transcription factors may further modulate the global activity of endogenous gene promoters regulated by multiple GR-responsive transcription factors. In addition, cell or tissue-specific effects might also influence transcriptional responses to CpdA. This phenomenon might explain why in fibroblasts only a marginal CpdA-mediated transrepression is observed for TNF-induced IL-6 (Figs. 1a, 2a), whereas in murine sera an enhanced IL-6 production is observed (Fig. 10c). The latter situation is of course the result of a combined effect on multiple tissues and circulating immune cells. In a recent study, CpdA could efficiently transrepress the *MMP13* (*collagenase*-*3*) gene in MEF cells [57]; and in primary synovial cells and osteoblast cells, as well as in spinal cord tissue, CpdA could transrepress IL-6 to the same extent as DEX [58]. However, in the same primary osteoblasts CpdA did fail, as expected, to efficiently suppress two other typical AP-1-driven genes, namely those coding for IL-11 and osteoprotegerin [58]. Literature reports confirm that Compound A can also negatively affect IL-6 gene expression and protein production in various tissues and cell lines with varying efficiencies [35, 59–61]. We therefore speculate that both the promoter context and the origin of the cell line or cellular tissue may co-determine differential responses towards CpdA as compared to classic GCs. Further studies in multiple cell systems are needed to unravel the underlying basis for these discrepancies.

Mechanistically, the paradoxical sustained AP-1 activity and inhibition of NF-κB activity by CpdA in fibroblasts, in contrast to repression of both AP-1 and NF-κB by DEX, might be explained by compound-specific effects on the MAPK activation profiles. Indeed, although CpdA efficiently blocks activated ERK in L929sA cells (Fig. 6), which corresponds with its inhibitory capacity on the downstream target NF-κB, it fails to inhibit JNK activation (Fig. 7a). On the contrary, in presence of CpdA, the activated JNK signal is even sustained and slightly enhanced (Fig. 7a). However, this prolongation and enhancement of JNK phosphorylation appears not to require the presence of GR (Fig. 8b). GCs are known to drive the expression of *dusp1*, coding for MKP-1, which can target the phosphorylation of JNK [51]. CpdA, however, does not readily support the upregulation of classic GRE-regulated genes (Online Resource 3c). This would also include the *dusp1* MAPK phosphatase, as demonstrated for L929sA fibroblasts (Fig. 8c), human A549 epithelial cells and in vivo in murine lung [38] and primary microglial and astrocyte cultures [61], a result that may well correlate with a prolonged JNK MAPK activation, as observed here. However, as the CpdA-treated and inflammatory stimulated conditions show a prolonged phosphorylation of JNK in comparison to the solvent-treated set of samples (Figs. 7a, 8b) additional effects should be at play here. Nevertheless, the presence of GR is essential to mediate the gene regulatory effect of DEX and CpdA in both L929sA fibroblasts (manuscript 8c-e) and A549 cells (Online Resource 7) for both IL6, IL8 and the AP-1 regulated c-Jun and MMP13.

Although the in silico virtual docking analysis of the group of Budunova [62] modeled CpdA in the GR ligand-binding pocket, we currently cannot exclude other modes of binding as the CpdA-bound GR structure has not yet been crystallized, or out-of-target effects. In that context, we recently observed that CpdA was able to efficiently block the TNF-induced phosphorylation of all three MAPK, p38, ERK, and JNK in primary human synovial fibroblasts in a GR-independent manner [63]. Hence, a different cellular context is able to promote an entirely different outcome, with respect to MAPK regulation.

It has been reported before that DEX-activated GR is recruited onto AP-1-dependent promoters, as such contributing to transrepression [64, 65]. In accordance with the gene and protein expression analyzes of c-Jun, ChIP analyses revealed that only DEX-activated GR but not CpdA-activated GR was retrieved on the *c*-*jun* gene promoter (Fig. 9a). Nevertheless, both DEX and CpdA translocate cytoplasmic GR to the nucleus [35]. Furthermore, and in agreement with the findings of Rogatsky et al. for *MMP*-*13 (collagenase*-*3)* in UO2S cells [65], we could verify that the occupation by c-Jun of the *MMP13* (*collagenase*-*3*) or *c*-*jun* gene promoters was constitutive and unchanged following either activating or repressing conditions (data not shown). Based on the ChIP data for the *c*-*jun* gene promoter (Fig. 9a), it is tempting to speculate that on the IL-6 gene promoter the observed GR recruitment level may primarily reflect the recruitment onto the NF-κB site, since the close proximity between the NF-κB and AP-1 sites in the IL-6 gene promoter does not allow to distinguish recruitment onto either binding site alone.

We have described before that CpdA is a selective modulator of GR which favors monomeric GR formation and, as such, does not support classic GRE-mediated gene transcription [35, 56]. We have shown here that CpdA is also able to differentiate between NF-κB- and AP-1-dependent transrepression. As an important physiological consequence of marked differential targets in vivo, we observed an increased sensitivity towards TNF lethality with a CpdA treatment (Fig. 10b) as opposed to a treatment with DEX [30]. It is clear that in a hyperinflammatory context, CpdA is not able to inhibit IL-6 (as observed for fibroblasts in vitro), but on the contrary leads to an enhanced IL-6 protein production in vivo. In accordance with the in vitro data on JNK MAPK, and by using a JNK2^−/−^ mouse model, we established a direct involvement of activated JNK, more specifically of JNK2, in the CpdA-mediated shift toward increased sensitivity to TNF.

To the best of our knowledge, this is the first report on a non-steroidal GR modulator capable of discriminating between NF-κB and AP-1 signaling in different cellular contexts. Other groups have focused on identifying GR mutants with different transcription factor specificities [66–68]. Earlier, different GR surfaces and/or mechanisms have been proposed to be involved in the repression of NF-κB or AP-1, and cell-type restrictions have also been noted for the gene regulatory actions of GR point mutants [66]. The team of Okret characterized a GR point mutation in the DNA-binding domain (rat GR R488Q), which is able to distinguish between NF-κB and AP-1 repression, in this particular case favoring AP-1 transrepression [66]. Taken together, the present data support the notion that GR may utilize different mechanisms to repress NF-κB as compared to AP-1.

In conclusion, a ligand for GR that selectively targets NF-κB and not AP-1 signaling pathways, not only narrows down the number (quantity) of affected targets and biological processes but also the extent (quality) of affected targets and processes. Hence, a discriminating compound, such as CpdA, is a very attractive tool to deepen our understanding of NF-κB- and AP-1-selective GR responses in specific tissues. Given the surprising opposite effects to DEX in terms of survival in an aggressive model of systemic inflammation, our study additionally conveys the important message that GR modulators with a differential transcription factor targeting profile may potentially aggravate an inflammatory response instead of resolving it. It remains to be investigated further whether the development of novel compounds with a similar level of selectivity may hold therapeutic promise, in specific clinical settings.

### Electronic supplementary material

Below is the link to the electronic supplementary material.

Supplementary material 1 (PDF 203 kb)

## Abbreviations

AP-1: Activator protein 1
β-gal: β-Galactosidase
CpdA: Compound A
CREB: cAMP-responsive element binding protein
DEX: Dexamethasone
DUSP1: dual-specificity phosphatase 1
ERK: Extracellular signal-regulated kinase
GC: Glucocorticoid
GILZ: GC-induced leucine zipper
GR: Glucocorticoid receptor
GRE: GC response element
H: Histone
IκB: Inhibitor of NF-κB
IL: Interleukin
i.p.: Intraperitoneally
IPA: Ingenuity pathway analysis
luc: Luciferase
JNK: Jun N-terminal kinase
MAPK: Mitogen-activated protein kinase
MCP1: Monocyte chemoattractant protein 1
MEF: Mouse embryonic fibroblast
MMP: Matrix metalloproteinase
MSK: Mitogen- and stress-activated protein kinase
NF-κB: Nuclear factor-κB
PBS: Phosphate-buffered saline
SGRM: Selective GR modulator
STS: Staurosporine
TNF: Tumor necrosis factor
TPA: 12-*O*-tetradecanoylphorbol-13-acetate
