# Supplementary material for: Selective modulation of the glucocorticoid receptor can distinguish between transrepression of NF-κB and AP-1
Source: Cell Mol Life Sci. 2013 Jun 20;71(1):143–63. doi: 10.1007/s00018-013-1367-4 (PMC3889831; doi:10.1007/s00018-013-1367-4)
Supplement: Supplementary file 1 — Supplementary material 1 (PDF 203 kb) [file 18_2013_1367_MOESM1_ESM.pdf]

Selective modulation of the Glucocorticoid Receptor can distinguish between transrepression of NF-κB and AP-1

Ilse M. Beck[1,2, ‡], Karolien De Bosscher[1,3, ‡,§], Lien Dejager[4], Nadia Bougarne[1,3], Anthoula Gaigneaux[5], Sébastien Chateauvieux[5], Dariusz Ratman[3], Marc Bracke[2], Jan Tavernier[3], Wim Vanden Berghe[1,6], Claude Libert[4],Marc Diederich[7] and Guy Haegeman[1,8,§].

[1] Laboratory of Eukaryotic Gene Expression & Signal Transduction (LEGEST), Department of Physiology, Ghent University, Belgium

[2] Laboratory of Experimental Cancer Research Department Radiation therapy & Experimental Cancer Research, Ghent University Hospital, Belgium

[3] Cytokine Receptor Lab, VIB Department of Medical Protein Research, Ghent University, Belgium

[4] Department for Molecular Biomedical Research, VIB, Ghent, Belgium and Department of Biomedical Molecular Biology, Ghent University, Ghent, Belgium

[5] Laboratoire de Biologie Moléculaire et Cellulaire du Cancer (LBMCC), Hôpital Kirchberg, Luxemburg, Luxemburg

[6] Lab Protein Chemistry, Proteomics & Epigenetic Signaling (PPES), Department Biomedical Sciences, University of Antwerp, Belgium

[7]Department of Pharmacy, College of Pharmacy, Seoul National University, Seoul 151-742, S. Korea

[8] present address: Division of Molecular Medicine, Department of Research and Development, Faculty of Medicine, Siriraj Hospital, Mahidol University, Bangkok 10700, Thailand

[‡] These authors contributed equally

[§] Correspondence to Karolien De Bosscher, Cytokine Receptor Lab, VIB Department of Medical Protein Research, VIB, UGent, Albert Baertsoenkaai 3, B-9000 Gent, Belgium; Tel +3292649363; Fax: +3226499490, e-mail: Karolien.DeBosscher@vib-ugent.be
or Guy Haegeman, Department of Physiology, LEGEST, Proeftuinstraat 86 – INW – N1 first floor, B-9000 Gent, Belgium, Tel +3292645166, Fax +3292645304, e-mail: Guy.Haegeman@ugent.be

Running title: Selective transcription factor cross-talk by GR

IMB&KDB_Online Resource_1

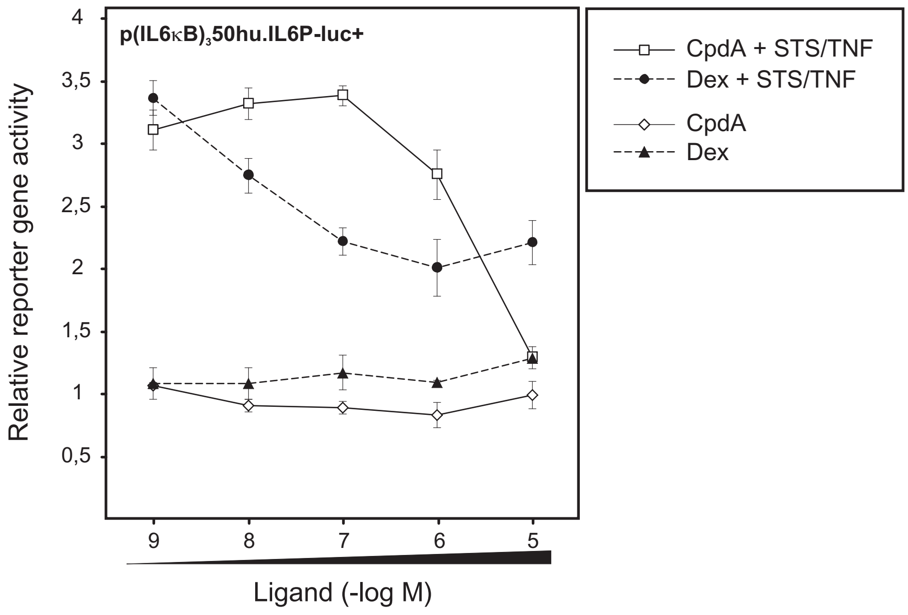

Online Resource 1

CpdA-mediated transrepression of the IL-6 gene promoter in fibroblast cells is only efficient in absence of a functional AP-1-response element

Subconfluent L929sA cell monolayers stably transfected with the p(IL6κB)$_3$50hu.IL6P-luc+ reporter gene construct were grown in 24-well plates. Cells were pretreated for 1h, using DEX or CpdA with increasing concentrations, as indicated, followed by a treatment with solvent or TNF (2000IU/ml) and STS (60nM), for5 h. At the end of the induction, cell lysates were assayed for reporter gene activities. Total solvent concentration was kept similar in all conditions. The experiments are carried out in triplicate. Results are shown +/-SD and are representative of three independent experiments.

IMB&KDB_Online Resource_2

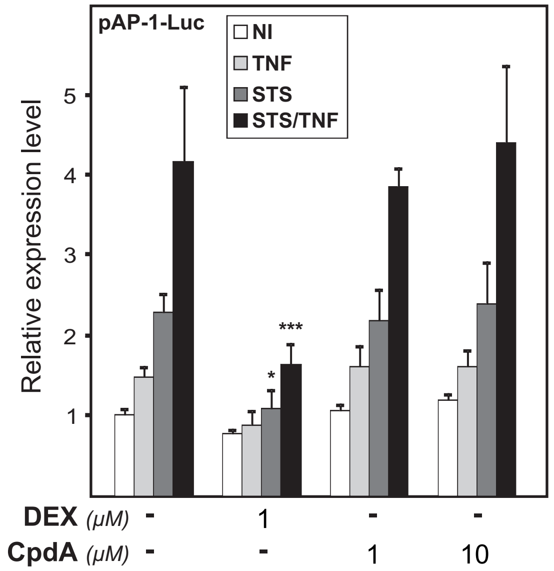

Online Research 2

AP-1-driven gene transcription is downregulated by DEX but not by CpdA

Subconfluent L929sA cell monolayers, stably transfected with the pAP-1-Luc reporter gene construct, were untreated or treated with 2000IU/ml TNF, with 60nM STS, with 50ng/ml TPA or a combination hereof, for 5h, in the presence or not of DEX (1μM) or CpdA (1 or 10μM*)*, starting at 1h. At the end of the induction, cell lysates were assayed for reporter gene activities. The experiment is carried out in triplicate. Results are shown +/-SD and are representative of at least two independent experiments. Total solvent concentration was kept similar in all conditions. *p<0,05, *** p< 0,001. Comparisons were made versus the respective pro-inflammatory stimuli.

IMB&KDB_Online Resource_3

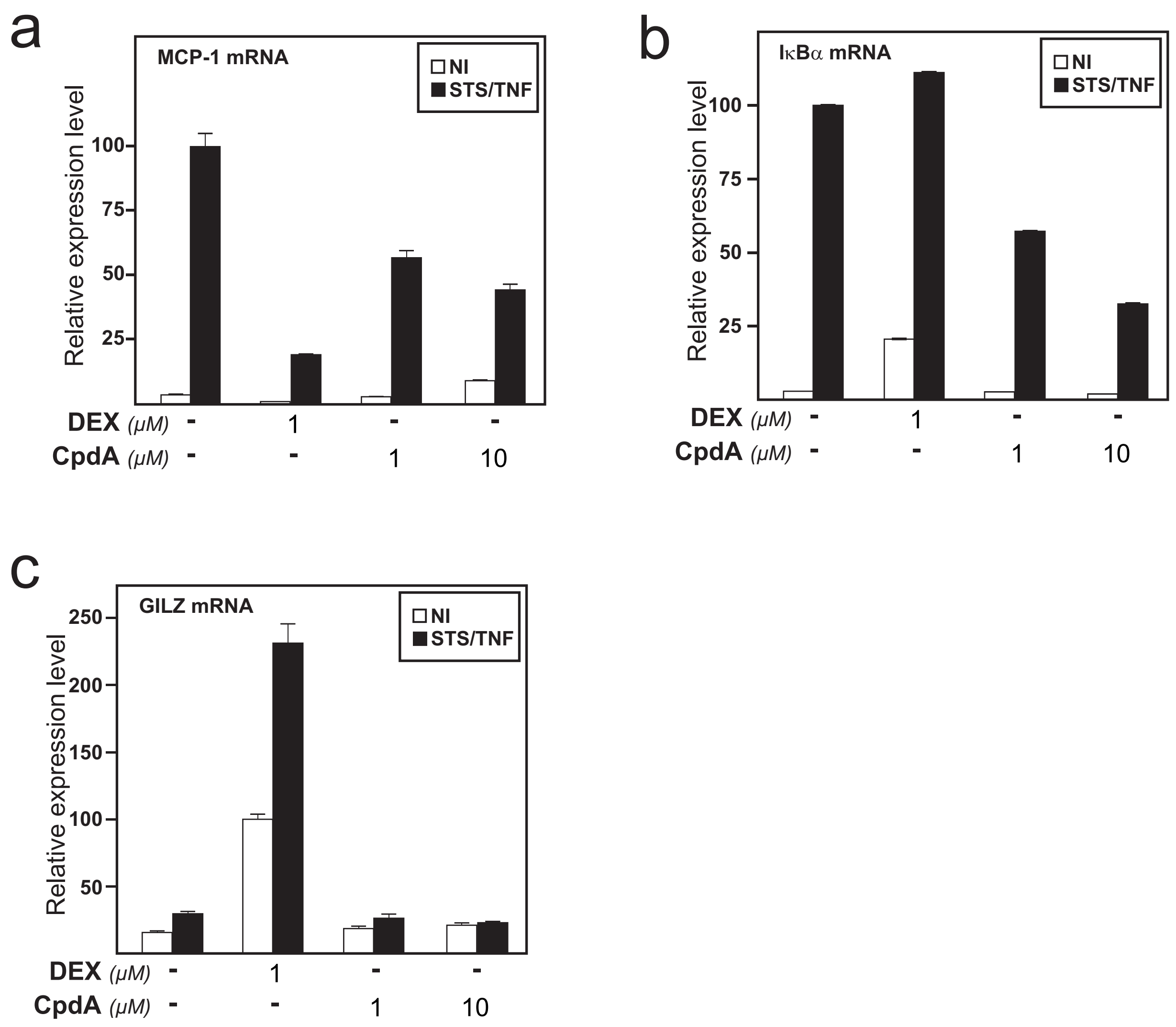

Online Resource 3

DEX and CpdA instigate differential effects, depending on the promoter context

L929sA cells, starved for 48h in DMEM devoid of serum, were pre-incubated with solvent, DEX (1µM), or CpdA (1 or 10µM) for 1h, before STS (60nM) and TNF (2000IU/ml) were added, where indicated, for 6h. Total RNA was isolated and subjected to RT-qPCR assaying the mRNA levels of MCP-1, IκBα, and GILZ and two household gene mRNA levels. Specific signal for cDNA of MCP-1 (a), IκBα (b), and GILZ (c) was normalized to the averaged household genes signal. The STS/TNF condition was set as 100 and all other conditions were recalculated accordingly to allow ratio comparisons. Total solvent concentration was kept similar in all conditions. Results are shown +/-SD. The experiment was carried out in triplicate, and the result is representative for at least two independent experiments.

IMB&KDB_Online Resource_4

422 genes
TNF inducible

MARCH3	C7orf49	CXCR3	GBP2	JUN	NINJ1	PTP4A3
ABTB2	C8orf4	CXCR4	GBP3	KIAA1217	NKD2	PTPN1
ADAR	C9orf30	CXorf38	GBP4	KIAA1609	NKX3-1	PTX3
ADORA2A	CA8	CYLD	GCH1	KIAA1949	NOD2	RAB9A
ADRB2	CAMK1G	CYP26B1	GFPT2	KIF1B	NPHS1	RAB9BP1
AKAP13	CARD14	CYP2C9	GJB2	KIRREL2	NPTX1	RAPH1
AKAP2	CARKD	CYTH1	GTF3C4	KLF6	NR4A1	RARRES1
AMPD3	CASP10	CYTH4	HBEGF	KREMEN2	NR4A2	RASGEF1A
ANK2	CASP7	DAPK2	HCP5	LAD1	NR4A3	RASSF5
ANKRD33B	CBFA2T3	DCUN1D3	HEPACAM2	LAMB3	NRG1	RCAN1
APOL2	CBR3	DDX10	HINT3	LAMC2	NUAK2	RELA
APOL3	CCDC130	DENND2D	HIVEP1	LIF	NUB1	RELB
APOL4	CCL17	DENND5A	HIVEP2	LOC284570	NUP153	RELT
APOL6	CCL2	DRAM1	HLA-A	LOC286359	OLR1	REPIN1
ASS1	CCL20	DTX3L	HLA-C	LOC440934	OSGIN2	RFFL
ATF3	CCL4	DUSP10	HLA-F	LOC646626	OVOS2	RFTN1
B2M	CCL5	DUSP16	HLA-J	LOC728743	P4HA2	RFX2
B4GALT1	CCRN4L	DUSP5	HNRNPC	LOC80154	PARP8	RHCG
BAHCC1	CD274	DUSP5P	HS3ST1	LRIG1	PBX4	RHOG
BAMBI	CD40	DUSP6	ICAM1	LRRC32	PDE4B	RIPK2
BATF3	CD83	DUSP8	ICAM4	LTB	PDE9A	RND1
BBC3	CDC42EP2	EBI3	ICAM5	MAFF	PDGFB	RNF144B
BCL2A1	CDC42EP4	EFNA1	IDO1	MAMLD1	PFKFB4	RNF24
BCL2L11	CEBPB	EFNA3	IFIT2	MAP2K3	PHC2	RRAD
BCL3	CEBPD	EFNB1	IFIT3	MCL1	PHF15	RTP4
BCL9L	CELF1	EGFR	IFNAR2	MESDC1	PIF1	S100A3
BDKRB1	CFB	EHD1	IFNGR2	MFHAS1	PIM1	SAMD4A
BDKRB2	CFLAR	EIF2C2	IKBKE	MICAL2	PIM3	SAT1
BHLHE41	CH25H	ENGASE	IL10RB	MICB	PISD	SDC4
BID	CHST2	EREG	IL15	MMP19	PLAT	SELE
BIK	CIDECP	ETS1	IL15RA	MOBKL2C	PLAU	SEMA4C
BIRC3	CITED4	ETV6	IL1A	MREG	PLAUR	SERPINA3
BPGM	CLDN1	EXT1	IL1B	MSC	PLEKHA4	SERPINB8
BTG3	CLDN5	FAM110A	IL23A	MSX1	PLEKHG2	SERPINB9
BTN2A1	CLEC2D	FAM129A	IL27RA	MYBPC1	PLEKHG3	SFTPC
BTN2A2	CLEC4E	FBLN5	IL32	MYEOV	PLK2	SH2B3
BTN2A3	CLIC4	FGF18	IL3RA	N4BP3	PML	SH2D1B
C10orf10	CLIP2	FJX1	IL4I1	NAB1	PNMA2	SH3PXD2A
C11orf68	CREB3L3	FLJ36031	IL4R	NACC1	PNRC1	SHB
C12orf34	CSF1	FOSB	IL6	NAV3	POM121L9P	SLA2
C15orf37	CSF2	FOSL1	IL7R	NCOA7	POU5F1	SLC12A7
C15orf48	CSF3	FOSL2	IL8	NEDD4L	PPAP2B	SLC25A28
C1QTNF1	CTNNB1	FOXO1	INHBA	NEURL3	PPARD	SLC25A37
C20orf54	CTSC	FSTL3	IRAK2	NFIL3	PPIF	SLC26A9
C21orf121	CX3CL1	FZD9	IRF1	NFKB1	PPP1R15B	SLC2A6
C3	CXCL1	G0S2	ISG20	NFKB2	PRDM1	SLC31A2
C3orf52	CXCL10	GABARAPL1	ITGA5	NFKBIA	PRR5	SLC37A1
C3orf59	CXCL2	GABPB1	ITPKC	NFKBIB	PSMA6	SLC6A12
C5orf56	CXCL3	GATA6	JAG1	NFKBID	PTAFR	SLFN5
C6orf150	CXCL6	GBP1	JAK2	NFKBIE	PTGS2	SLPI

Online Resource 4

Whole genome transcriptome analysis of A549 cells in response to TNF, DEX and/or CpdA

A549 cells, starved in 0% DMEM for 24h, were pretreated for 1h either with DEX (1μM) or CpdA (10μM), either or not followed by 3h treatment with TNF (2000IU/ml). Gene expression levels of corresponding RNA samples were evaluated by an Agilent array. Genes with adjusted p-values lower than 0.05 in at least one contrast and a folchange higher than 1.3 were selected as significant.

Of 422 TNF inducible genes, 179 genes were repressed by DEX, of which 121 genes were exclusively repressed by DEX but not CpdA and 58 were repressed by both CpdA and DEX.

IMB&KDB_Online Resource_4_continued

		121 genes			50 genes	58 genes	
**TNF inducible, continued**		**TNF+DEX repressed**			**TNF+CpdA repressed**	**TNF+DEX+CpdA repressed**	
SMAD3	UBD	ADAR	HBEGF	SLFN5	ADORA2A	ANK2	SPEN
SMG1	UBE2H	APOL3	HINT3	SMAD3	ASS1	BAHCC1	SRCIN1
SMOX	UNC5B	APOL6	HIVEP1	SSH1	BCL9L	BPGM	TBC1D10A
SMURF1	UNKL	ATF3	HNRNPC	STYK1	C12orf34	C20orf54	TNF
SMURF2	USP31	B2M	IDO1	TIAM2	C1QTNF1	CCL2	TNFAIP6
SNAPC4	USP42	BBC3	IFIT2	TIFA	C3	CCL5	TRAF1
SOCS1	USP43	BCL2A1	IL15RA	TMEM158	C7orf49	CD83	USP43
SOD2	VCAM1	BCL2L11	IL23A	TNFRSF10B	CAMK1G	CLIP2	VCAM1
SOX9	VDR	BCL3	IL4I1	TNFRSF9	CBFA2T3	CX3CL1	
SP100	VEGFC	BDKRB1	IL6	TP63	CCL17	DUSP10	
SPEN	WTAP	BDKRB2	IL8	TRIML2	CD40	EXT1	
SPRY4	XBP1	BHLHE41	ISG20	UBD	CEBPD	FOSL1	
SRCIN1	YWHAG	C11orf68	JAG1	UBE2H	CFB	G0S2	
SRSF12	ZBTB46	C15orf48	JUN	UNKL	CREB3L3	GBP4	
SSH1	ZBTB7A	C5orf56	KIAA1217	USP42	CSF3	HEPACAM2	
SSTR2	ZC3H12A	C6orf150	LAMB3	VDR	EBI3	IFIT3	
ST3GAL4	ZFP36	C8orf4	LIF	VEGFC	FGF18	IL1A	
STAT5A	ZKSCAN1	CASP10	MAMLD1	YWHAG	FSTL3	IL1B	
STX11	ZNF101	CASP7	MAP2K3	ZKSCAN1	FZD9	IL3RA	
STYK1	ZNF641	CBR3	MFHAS1	ZNF641	HLA-F	JAK2	
TAP1	ZNF697	CCRN4L	NAB1	ZNF697	HLA-J	KIF1B	
TAPBP	ZNFX1	CD274	NAV3		ICAM1	KIRREL2	
TBC1D10A		CELF1	NCOA7		IFNAR2	LOC286359	
TBC1D2B		CHST2	NEURL3		IFNGR2	LRRC32	
TFF1		CITED4	NFKB1		IKBKE	LTB	
THSD1		CLDN1	NR4A1		IL32	MSC	
TIAM2		CLEC2D	NR4A2		IRF1	MYBPC1	
TIFA		CSF2	NR4A3		ITGA5	NFKB2	
TMEM158		CTSC	NRG1		KIAA1949	NKX3-1	
TMEM88		CXCL1	OSGIN2		LOC728743	NPHS1	
TNF		CXCL3	OVOS2		MYEOV	OLR1	
TNFAIP1		CXCL6	PDGFB		NINJ1	PDE4B	
TNFAIP2		CXCR4	PIM1		NOD2	PIF1	
TNFAIP3		CXorf38	PLAU		PDE9A	PLAT	
TNFAIP6		CYP26B1	PLEKHA4		PISD	PLAUR	
TNFRSF10B		DDX10	PLK2		PTP4A3	PNMA2	
TNFRSF9		DENND5A	PPARD		RELA	PPP1R15B	
TNFSF14		DTX3L	PPIF		RHOG	PTAFR	
TNIP1		DUSP16	PRR5		RRAD	RASSF5	
TP63		DUSP6	PTGS2		SERPINA3	RTP4	
TRAF1		DUSP8	PTX3		SLC2A6	SDC4	
TRAF2		EFNA1	RFFL		SPRY4	SH2B3	
TRAF3		EHD1	RFX2		SSTR2	SH3PXD2A	
TRIM47		EREG	RIPK2		STAT5A	SHB	
TRIM56		ETV6	RND1		TNFAIP2	SLA2	
TRIM8		FAM129A	SERPINB8		TNIP1	SLC6A12	
TRIML2		FOSB	SERPINB9		TRIM47	SMG1	
TUBB2A		GBP1	SFTPC		UNC5B	SMOX	
TUBB2B		GBP2	SLC12A7		ZBTB7A	SMURF2	
TYK2		GBP3	SLC25A37		ZNFX1	SOX9	

Online Resource 4

Whole genome transcriptome analysis of A549 cells in response to TNF, DEX and/or CpdA

A549 cells, starved in 0% DMEM for 24h, were pretreated for 1h either with DEX (1µM) or CpdA (10µM), either or not followed by 3h treatment with TNF (2000IU/ml). Gene expression levels of corresponding RNA samples were evaluated by an Agilent array. Genes with adjusted p-values lower than 0.05 in at least one contrast and a folchange higher than 1.3 were selected as significant.

Of 422 TNF inducible genes, 179 genes were repressed by DEX, of which 121 genes were exclusively repressed by DEX but not CpdA and 58 were repressed by both CpdA and DEX.

IMB&KDB_Online Resource_5

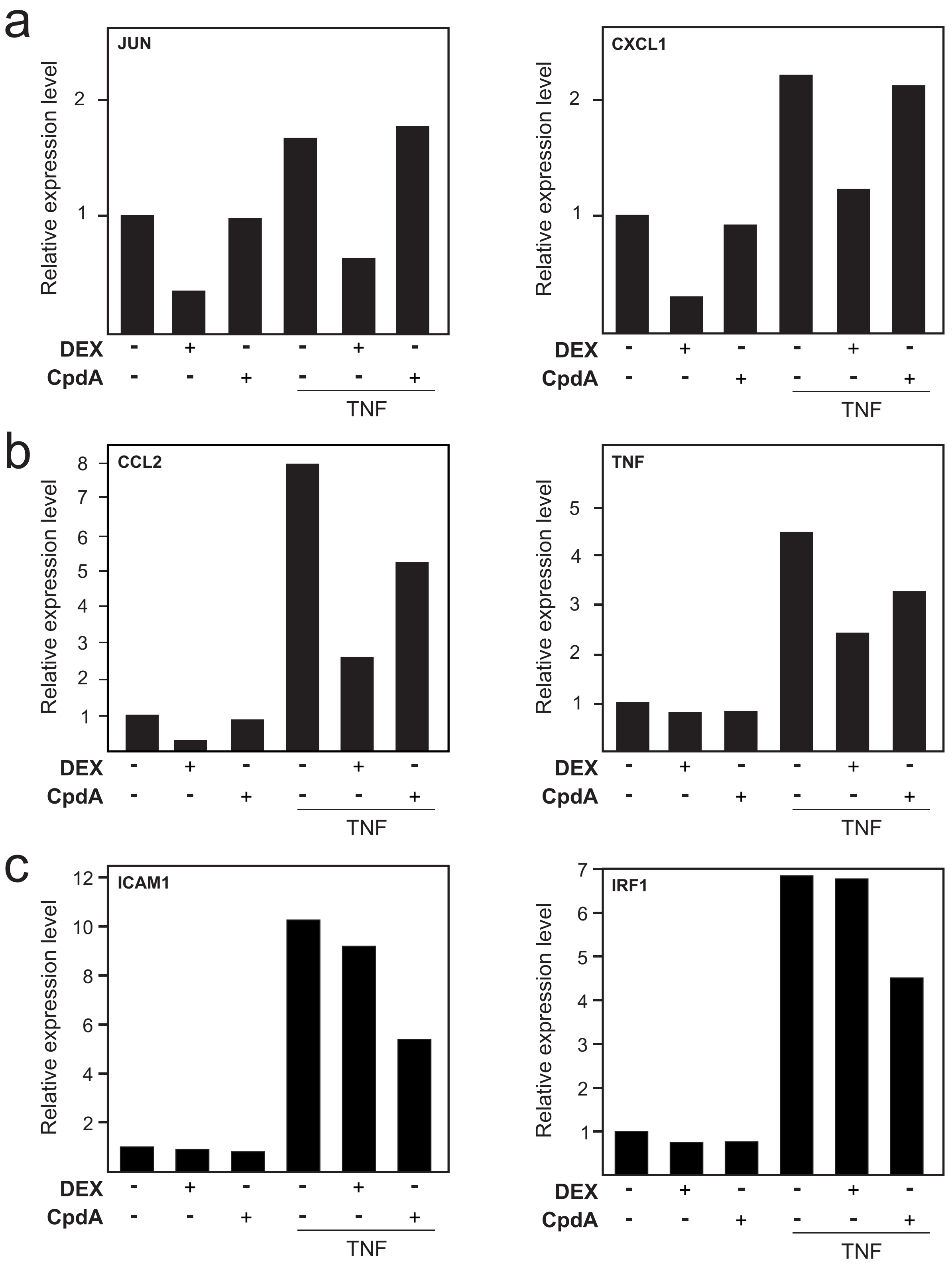

Online Resource 5

Whole genome transcriptome analysis of A549 cells in response to TNF, DEX and/or CpdA

A549 cells, starved in 0% DMEM for 24h, were pretreated for 1h either with DEX (1µM) or CpdA (10µM), either or not followed by 3h treatment with TNF (2000IU/ml). Gene expression levels of corresponding RNA samples were evaluated by an Agilent array. Genes with adjusted p-values lower than 0.05 in at least one contrast and a folchange higher than 1.3 were selected as significant. Gene expression profiles of select targets are exemplarly displayed. *(a)* Selected gene expression responses repressed by DEX, but not or to a lesser extent by CpdA. *(b)* Selected gene expression responses repressed by both DEX and CpdA. *(c)* Selected gene expression responses repressed by CpdA, but not or to a lesser extent by DEX.

IMB&KDB_Online Resource_6

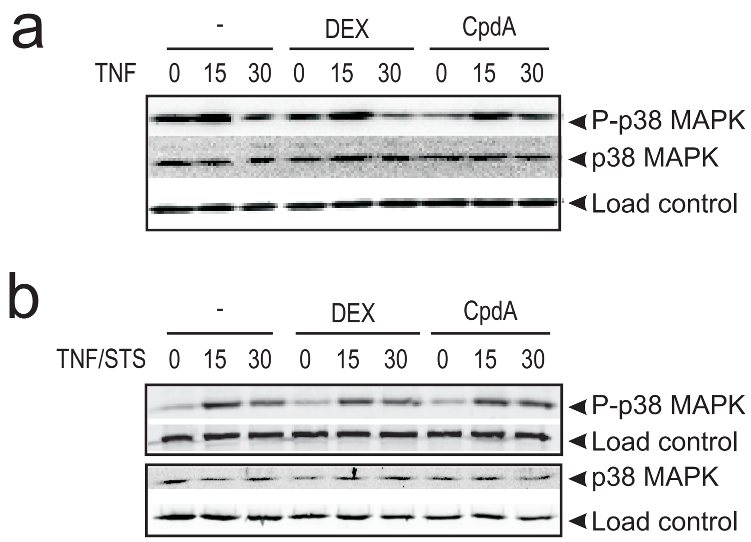

Online Resource 6

DEX nor CpdA affect p38 MAPK phosphorylation, irrespective of the stimulus

(a) and *(b)* L929sA cells, starved for 48h in DMEM devoid of serum, were pretreated with solvent, 1µM DEX, or 10µM CpdA for 1h, followed by either or not TNF (2000IU/ml) (panel a), or TNF combined with STS (60nM) (panel b), for the indicated time points (in minutes). Cell lysates were made and activated p38 MAPK was detected using the phospho-specific p38 MAPK antibody. Aspecific bands, non-phosphorylated proteins and/or NF-κB p65 served as a loading control (indicated as Load control).

IMB&KDB_Online Resource_7

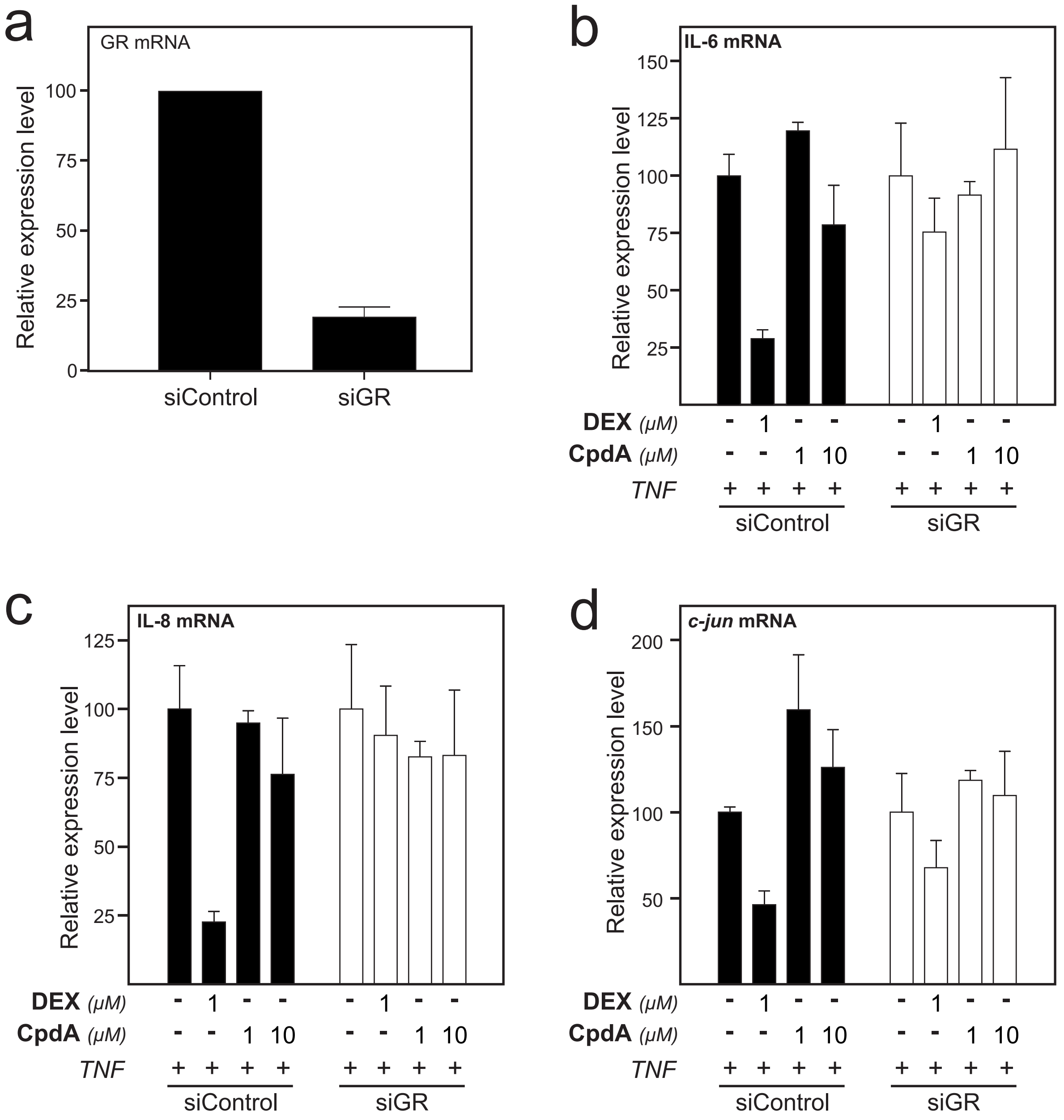

Online Resource 7

GR is essential to mediate the effect of DEX and CpdA on gene expression in A549 cells

A549 cells were transfected with siRNA control (siControl) or siRNA targeted at GR (siGR) and were allowed to rest for 24h. Subsequently, cells were pre-incubated with solvent, DEX (1µM), or CpdA (1 or 10µM) for 1h, before TNF (2000IU/ml) was added for 24h. Total RNA was isolated and subjected to RT-qPCR for specific target genes, and expression levels in each treatment group were normalized to the values in the TNF-stimulated samples. In *(a)*, the expression levels for GR (siControl) were set at 100 and the siGR condition was recalculated accordingly. Normalized mRNA levels for IL-6 *(b)*, IL-8 *(c)* and *c-Jun (d)* was presented as induction fold with the solvent condition of both siControl and siGR set as 100. Total solvent concentration was kept similar in all conditions. Results are shown +/-SD. The experiment was carried out in duplicate.
